# Exploring the Mechanism of Resveratrol in Reducing the Soft Tissue Damage of Osteoarthritis Based on Network Pharmacology and Experimental Pharmacology

**DOI:** 10.1155/2021/9931957

**Published:** 2021-10-04

**Authors:** Zhiyong Long, Wang Xiang, Jun Li, Tiejun Yang, Ganpeng Yu

**Affiliations:** ^1^Shantou University Medical College, Shantou University, Shantou, Guangdong, China; ^2^The Affiliated Hospital of Guilin Medical University, Guilin, Guangxi Province, China; ^3^People's Hospital of Ningxiang City, Ningxiang City, Hunan Province, China

## Abstract

**Aim:**

To explore the mechanism of resveratrol in reducing the soft tissue damage of osteoarthritis (OA) based on network pharmacology.

**Methods:**

Pharmmapper was used to predict the target of resveratrol, OMIM and Genecards were used to collect OA-related disease genes, and David ver 6.8 was used for enrichment analysis. Then, animal experiments were carried out for verification. The rat OA model was established and the rats were randomly divided into 4 groups: model group, resveratrol low-dose group, resveratrol high-dose group, and blank control group for follow-up experiments. Hematoxylin-eosin (HE) staining was used to detect the degree of pathological damage of rat bones and joints. Enzyme-linked immunosorbent assay (ELISA) was used for the content of inflammatory factors. Western blot was used to detect the expression of Toll-like receptor 4 (TLR4), Myeloid differentiation factor 88 (MyD88), nuclear factor kappa B protein (NF-*κ*B), cysteine protease-9 (CASP-9), Bcl-2 protein, and Bax protein.

**Results:**

Through network pharmacological analysis, this study found that resveratrol may regulate the TLR4 signaling pathway, PI3K-Akt signaling pathway, FoxO signaling pathway, Osteoclast differentiation, Rheumatoid arthritis, etc. Animal experiments showed that compared with the model group, the pathological damage of bone and joint in the resveratrol low-dose and high-dose groups was significantly improved. Compared with the model group, the serum levels of IL-1beta, IL-6, IL-17, TNF-*α*, and MCP-1 in the resveratrol low-dose and high-dose groups were significantly reduced (*P* < 0.05); protein levels of TLR-4, MyD88, and NF-*κ*B p65 were significantly reduced (*P* < 0.05); caspase-9 and Bax protein levels were significantly reduced (*P* < 0.05), and Bcl-2 was significantly increased (*P* < 0.05).

**Conclusion:**

Resveratrol may inhibit the activation of the TLR4-mediated NF-*κ*B signaling pathway and has a repairing effect on soft tissue damage in OA.

## 1. Introduction

Osteoarthritis (OA) is a degenerative joint disease characterized by progressive articular cartilage destruction, subchondral bone changes, osteophyte formation, and synovial inflammation [1]. The clinical symptoms are mainly joint pain, swelling, stiffness, and mobility disorders, which seriously affect the quality of life of patients [2–4]. In the past 20 years, the number of patients with OA has increased dramatically [5, 6]. It is estimated that the current prevalence of OA in people over 50 years old worldwide is as high as 10% to 20%, and in the next 30 years, its prevalence may double [7]. So far, the clinical treatment of OA is mainly to relieve pain and maintain joint function. At present, nonsteroidal anti-inflammatory drugs (NSAIDs) have been used as a routine treatment for OA, but their adverse events should not be underestimated [8]. Therefore, it is very important to explore the occurrence and development mechanism of OA and find safer and more effective treatments for the prevention and treatment of OA.

Resveratrol is a natural polyphenol plant compound with a symmetric diphenylethylene structure. Its content is high in the rhizomes of grapes, cranberries, peanuts, mulberries, and other plants and the traditional Chinese medicine Polygonum cuspidatum [9–11]. It has almost no toxic and side effects on the human body and has been proven to have anti-inflammatory, antitumor, antioxidant, and immune-regulating effects [12]. Studies have shown that resveratrol has anti-inflammatory and chondroprotective effects in a rabbit OA model induced by endotoxin (LPS) [13]. Hua et al. found that resveratrol can prevent sodium nitrosoferricyanide-induced OA-like inflammation [14]. However, the mechanism of resveratrol in the treatment of OA is still unknown.

**Figure 1:**
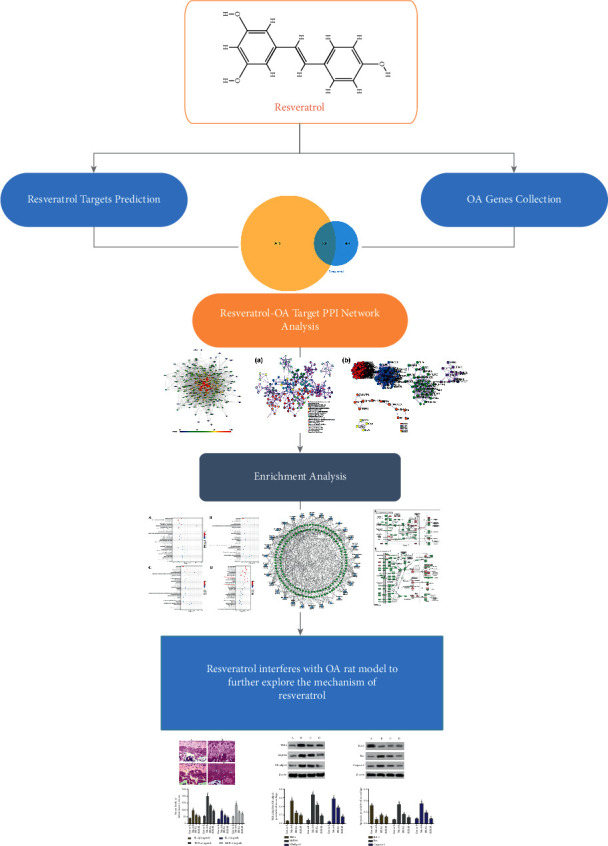
The idea and process of this research.

As a new interdisciplinary developed in recent years, network pharmacology has changed the previous drug discovery model of “single gene, single target, single disease.” The emergence of network pharmacology is of great significance for the discovery of new drugs and the multitarget research of drugs, and it is mainly used for drug toxicity prediction and drug readjustment indications [15, 16]. For different kinds of diseases, the location and characteristics of drug targets and disease genes in the network are different. Research shows that the distance between the drug target and the key node of the disease network has certain characteristics in the network. In addition, studies have found that in the process of rational drug design, the network distance between drug targets and corresponding disease genes has a tendency to become smaller and smaller [17]. This suggests that it is of great significance to probe the characteristics of drug targets in biological networks. Therefore, this study would explore the molecular network of resveratrol in the treatment of OA through network pharmacology and further verify the related signaling pathways and biological processes in OA rat models. The idea and process of this research are shown in Figure 1.

## 2. Materials and Methods

### 2.1. Potential Targets of Resveratrol Prediction and OA Disease Gene Collection

The relevant chemical information and several potential targets of resveratrol were searched through the PubChem database (https://www.ncbi.nlm.nih.gov) [18], and the chemical structure of resveratrol was drawn using ChemDraw 3D software and saved in sdf format. The SMILES structure of resveratrol was also collected from Pubchem. The “sdf” format of resveratrol was input into Pharmmapper (http://lilab-ecust.cn/pharmmapper/) to predict its potential targets [19]. The SIMES structure of resveratrol was input into Swiss Target Prediction (http://www.swisstargetprediction.ch/) [20], Similarity ensemble approach (SEA) (http://sea.bkslab.org/) [21], and STITCH Database (http://stitch.embl.de/) [22] to obtain the potential targets. The resveratrol targets were imported into UniProt (http://www.uniprot.org/) to obtain the official gene symbol. Finally, those targets were combined and deduplicated to obtain the set of resveratrol potential targets.

OA disease genes were searched and collected using the OMIM database (http://omim.org/) and Genecards (http://www.genecards.org) [23, 24]. The search results of Genecards and OMIM were merged and deduplicated to obtain OA-related genes set. The resveratrol targets and OA genes are shown in Table S1 (see supplementary materials). The potential target set of resveratrol and the OA target set were compared, and the overlapping part of the two was considered as the target of resveratrol in the treatment of OA (Table S2).

### 2.2. Resveratrol-OA Protein-Protein Interaction (PPI) Network Construction and Analysis

The String database (https://string-db.org/) is a database for searching protein interactions, including both direct physical interactions between proteins and indirect functional correlations [25]. The targets were input into String to collect the PPI data. Cytoscape 3.7.1 software was utilized for visualization. The node degree and betweenness centrality are used to reflect the importance of the node. The larger the value of the degree and the betweenness centrality, the more important the node in the network.

In order to further understand the functions of core target genes and the main pathways of resveratrol in the treatment of OA, the resveratrol-OA targets in the resveratrol-OA PPI network were input into David database (https://david.ncifcrf.gov/home.jsp) for Gene Ontology (GO) enrichment analysis and KEGG pathway enrichment analysis, and the species was selected as “Homo sapiens” [26].

### 2.3. Experimental Animals

Eighty (80) 6-week-old, SD male healthy rats were purchased from Guangdong Experimental Animal Center and raised in a standard environment, license number SCXK (Guangdong) 2018–0016. The weight of the rat is (180 ± 10) g. The rats were kept in a temperature-controlled (22°C ± 1°C) and light-controlled animal facility (200 lux, 12-hour light-dark cycle) for 7 days. The experiment was approved by the Animal Ethics Committee of Shantou University Medical College.

### 2.4. Reagents and Instruments

Resveratrol was purchased from the National Institute for the Control of Pharmaceutical and Biological Products (batch number: 110742200517, purity 99.9%). Collagenase Type II, (Cat. No.: C2-28, sigma company); BCA protein quantitative kit (batch number Q1220551), phenylmethylsulfonyl fluoride (PMSF, batch number: RE2173411), ethyl iodide Amide (IAA, batch number QL224490), RIPA high-efficiency lysate (batch number TJ272371), Ammonium bicarbonate powder (batch number BCBN6056 V) were purchased from Sigma Inc. The PVDF membrane was purchased from Millipore, USA (Cat. No. IPVH00010). Trypsin-EDTA digestion solution was purchased from Jiangsu KGI Biotechnology Co., Ltd. (Cat. No. KGY0012). HRP goat anti-rabbit secondary antibody IgG (Cat. No. ZB 2306) was purchased from Beijing Zhongshan Jinqiao Biotechnology Co., Ltd. The chemiluminescent substrate (Cat. No. A38555) was purchased from Thermo Company. IL-1*β* (H002), IL-6 (H007), IL -17 (H014), TNF-*α* (H052), and MCP-1 (H115) ELISA kits were purchased from Nanjing Jiancheng Institute of Bioengineering. Antibody TLR-4 (BA1717) and Antibody MyD88 (A00025-1) were purchased from Boster Biological Technology Co, Ltd. Antibody NF*κ*B p65 (ATA33904) was purchased from AtaGenix (Wuhan) Technology Co., Ltd. Antibody Caspase-9 (ab32539), antibody Bcl -2 (ab182858), antibody Bax (ab32503), and antibody *β*-actin (ab8226) were purchased from Abcam Inc. The desktop high-speed refrigerated centrifuge was purchased from Sigma Inc. The microplate reader was purchased from Thermo Company. The tissue disrupter was purchased from SPEX SamplePrep.

### 2.5. Animal Modeling, Grouping, and Intervention

The rats were randomly divided into a model group, resveratrol low-dose group, resveratrol high-dose group, and blank control group, with 20 rats in each group. The rats in the model group, resveratrol low-dose group, and resveratrol high-dose group were modeled [27, 28].

After the rats were anesthetized with 3% sodium pentobarbital (40 mg/kg), the right knee joint was disinfected with iodophor 3 times, and then the knee joint was punctured with a 1 mL syringe. 50 *μ*L of Type II collagenase (425 U/mg) with a concentration of 4 mg/mL was injected into the joint cavity. No other special treatment was given after the operation. On the 4th day, the above operation was repeated once, and the animals were raised freely for 3 days after making the model. The rats in the control group were injected with the same volume of normal saline at the same place and time point.

After the model was completed, the drug was started after 3 days of free breeding. Resveratrol low-dose group was given resveratrol 40 mg/kg and the resveratrol high-dose group was given resveratrol 80 mg/kg by gavage, twice daily for 4 weeks. The control group and the model group were given the same amount of normal saline intragastrically.

### 2.6. Collection, Preparation, and Observation of Knee Joint Specimens

After 4 weeks of intervention, the rats were fasted for 10 hours. After the rats were anesthetized by 3% sodium pentobarbital (40 mg/kg) intraperitoneal injection, about 4 mL of blood was taken from the abdominal aorta and placed in a 5 mL heparin sodium anticoagulation tube. After the blood was taken, the rats were sacrificed by neck dislocation. The entire knee joint was dissected and fixed with 4% paraformaldehyde for 24 h and decalcified with 15% ethylenediaminetetraacetic acid (EDTA) for 4 weeks. The cartilage was cut along the coronal surface of the joint, dehydrated by ethanol, transparent in xylene, embedded in paraffin, and sectioned. The thickness of the section was 7 *µ*m. The isolated cartilage tissue is used to detect protein inflammation and apoptotic protein expression by the Western Bolt method.

### 2.7. Pathological Observation

The articular cartilage sections were deparaffinized in xylene, then hydrated with gradient alcohol, and stained according to the instructions of the HE staining kit. The sections were stained in hematoxylin for 5 min, dyed in eosin staining solution for 2 min, soaked in distilled water, and then dehydrated in gradient alcohol in turn. Finally, it was made into pathological sections.

### 2.8. Detection of Serum Inflammatory Factors by ELISA

The rat blood was centrifuged at 3,000 r/min for 10 min, and the supernatant was taken. The antigen is dissolved in 50 mmol/L carbonate coating buffer at 4°C, 100 *µ*L/well is transferred to a 96-well microtiter plate, the antigen is coated overnight, and the coating solution is discarded. After washing, each well was blocked with 150 *µ*L of 1% BSA for 1 hour, washed with PBST 3 times, 100 *µ*L of serum with different dilution ratios was added, and control samples were added and incubated at 37°C for 2 hours. Then it was washed 5 times with PBST, 100 *µ*L of diluted HRP-labeled secondary antibody was added and incubated at 37°C for 1 h. After washing 5 times with PBST, the color developer was added to develop color for 20 min and measured with a microplate reader.

### 2.9. Detection of Inflammatory Factors by Western Blot

The rat cartilage tissue was prepared as a tissue homogenate, dissolved on ice for 25 min, centrifuged at 12,000 r/min for 10 min, and cell lysate containing protease inhibitors was added for total protein extraction. The BCA kit was used to determine protein content. An equal amount of protein sample (20 mg) was extracted and denatured at 100°C for 5 min. The proteins were then separated using SDS-PAGE gel electrophoresis and transferred to PVDF membranes. At 4°C, the PVDF membrane was added with corresponding monoclonal primary antibodies [TLR4 (1 : 200), MYD88 (1 : 500), NF-KBp65 (1 : 500), Caspase-9 (1 : 100), Bcl- 2, Bax (1 : 100), *β*-actin (1 : 500)] and incubated overnight. Then the horseradish peroxidase-labeled secondary antibody (1:2 000) was added at 4°C. The color was developed by the chemical substrate luminescence method. ChemiDoc XRS + System gel imager was used for image scanning analysis, Image-QuaNT software was used to measure its absorbance, and each *β*-actin was used as an internal reference to analyze the relative expression level.

### 2.10. Statistical Analysis

All the experimental data are statistically analyzed with the statistical software SPSS 20.0. The experimental results are expressed as mean ± standard deviation. An Independent *t*-test was used for pairwise comparison, and differences between groups were tested by one-way analysis of variance.

## 3. Results and Discussion

### 3.1. Resveratrol-OA PPI Network Analysis

**Figure 2:**
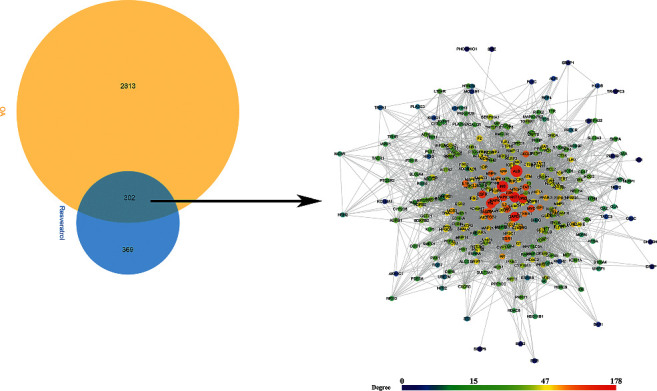
Venn diagram and Resveratrol-OA PPI network (the size of the node is positively correlated with its betweenness centrality.).

A total of 671 resveratrol potential targets and 3114 OA genes (3114 genes were searched from Genecards and 42 were searched from OMIM) were obtained. There are a total of 302 common targets in the resveratrol potential target set and OA gene set, which are considered as potential targets for resveratrol to treat OA. Among the 302 Resveratrol-OA targets, 297 can interact with each other. Hence, these 297 were used to construct the Resveratrol-OA PPI network (Figure 2).

### 3.2. Enrichment Analysis of Resveratrol-OA Targets

**Figure 3:**
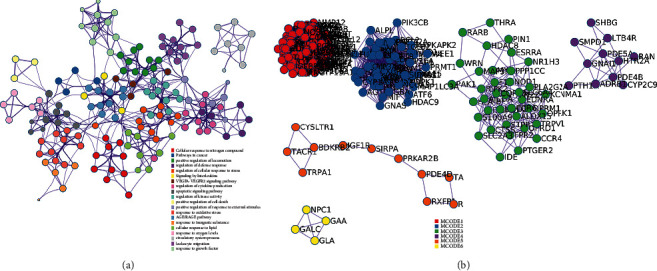
Visualizations of functional enrichment and interactome analysis result. (a) PPI network colored by cluster; (b) clusters.

**Figure 4:**
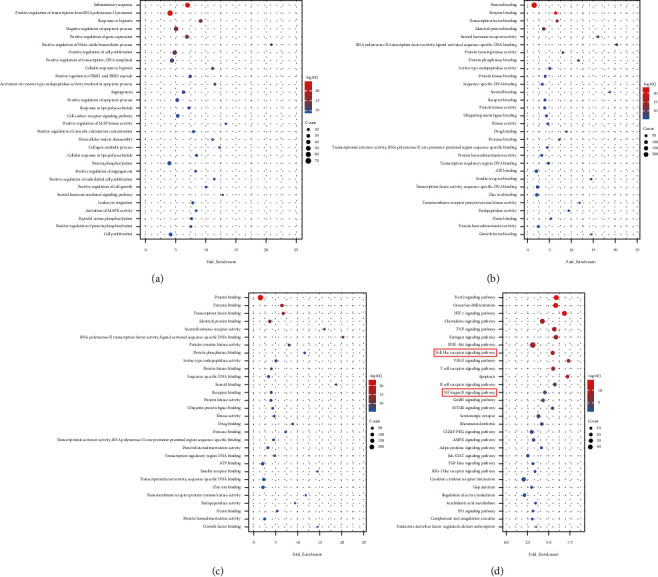
Bubble chart of enrichment analysis results. (a) biological processes; (b) cell components; (c) molecular function; (d) signaling pathways. *X*-axis stands for fold enrichment..

**Figure 5:**
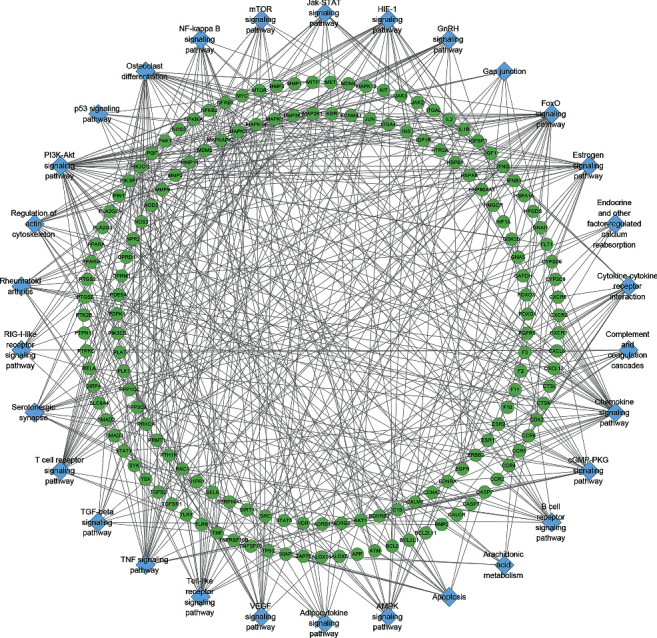
Signaling pathway-target network. Blue diamond stands for signaling pathway. Green circles stand for Resveratrol-OA targets.

**Figure 6:**
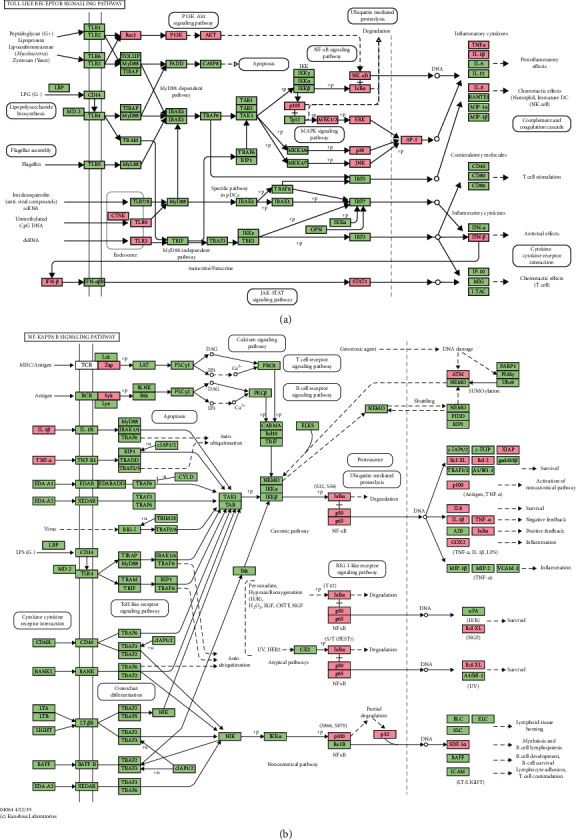
(a) Toll-like receptor signaling pathway adapted from KEGG (ID: hsa04620); (b) NF-kB signaling pathway adapted from KEGG (ID: hsa04064).

The 297 Resveratrol-OA targets in Resveratrol-OA targets PPI network were input into David for enrichment analysis. In the enrichment results of FDR<0.05, the biological processes, cell components, molecular functions, and signal pathways that may be related to OA were screened. Finally, a total of 171 OA-related biological processes, 43 cell components, 90 molecular functions, and 30 signal pathways were obtained (Table S3 and Figure 3). The top 30 biological processes, cell components, molecular functions, and signaling pathways are shown in Figure 4. The relationship between signaling pathways and targets is shown in Figure 5. The targets and genes in the Toll-like receptor signaling pathway and NF-kB signaling pathway are shown in Figure 6 as an example (the Resveratrol-OA targets were marked in pink) [29].

OA is a common degenerative disease that plagues middle-aged and elderly people. The degeneration of articular cartilage caused by the degradation of the cartilage extracellular matrix is the main pathological change of osteoarthritis. The extracellular matrix is mainly composed of type II collagen and Aggrecan. Mature chondrocytes can synthesize and secrete extracellular matrix, which plays a key role in maintaining the dynamic balance between extracellular matrix anabolic and catabolism [30]. Matrix metalloproteinases (MMPs) play an important role in the degeneration of osteoarthritis. MMP-13 is the most effective type II collagen degrading enzyme [31]. In OA pathological process, the secretion of MMP-13 increases and destroys Aggrecan and type II collagen in the extracellular matrix, which ultimately leads to cartilage degeneration and destruction [32].

In the OA model induced by medial meniscus instability surgery, MMP-13 knockout mice inhibited the development of OA by protecting cartilage from proteoglycan loss and structural damage [33]. In clinical samples, MMP-13 is abnormally expressed at different stages of OA: it is upregulated in the cartilage of patients with OA in the early stage and downregulated in the late stage [34]. In addition, MMP-13 is a central node in the cartilage degradation network [35], and its activity can be regulated at multiple levels such as transcription, epigenetic changes, and autophagy [36, 37]. The intervention of resveratrol can decrease the expression of MMP-13, Nuclear factor kappaB (NF-kB) and other inflammatory factors closely related to OA, such as Interleukin-6 (IL-6), Cyclo-oxygenase-2 (COX-2) [38], IL-1*β* [39], etc. However, the mechanism of resveratrol's anti-OA effect has not been elucidated. Studies have shown that OA may be related to chronic low-grade inflammation, and the expression of a variety of cytokines and inflammatory mediators in the OA state is significantly increased [40], and these inflammatory responses may be related to TLR4. TLR4 can induce synovial cells, chondrocytes, etc., to secrete and release IL-1*β* and other inflammatory factors, which play a key role in the pathogenesis of OA. The study also found that the expression of TLR4 in OA chondrocytes induced by IL-1*β* increased significantly, but after treatment with resveratrol, its expression decreased significantly. Therefore, it is believed that the occurrence and development of OA may be related to the activation of the TLR4 signaling pathway [41], and resveratrol can exert anti-OA effects by inhibiting the Toll-like (TLR4) signaling pathway [42].

TLR4 is one of the important members of the TLRs family and plays a vital role in the process of inducing inflammation [38]. At present, studies have shown that resveratrol can exert anti-OA effects by inhibiting TLR4/MyD88-dependent and independent signaling pathways [43]. Other studies have shown that resveratrol can also exert anti-inflammatory effects through the PI3K/Akt signaling pathway in macrophages [44]. PI3K is an important member of the phospholipid kinase family [45, 46]. Akt is an important direct downstream molecule of PI3K, and activation of PI3K can directly promote Akt phosphorylation and activation. The activation or inhibition of Akt can directly act on its downstream signal molecules and then play a role in regulating cell proliferation, apoptosis, or other important physiological processes [47]. It has been reported in the literature that when LPS acts on human pancreatic cancer cells, it can upregulate the expression of PI3K and Akt. But after adding TLR4 siRNA to silence TLR4, the expression of TLR4 and p-Akt decreased significantly, and after silencing TLR4 and then stimulated by LPS, the expression of TLR4 and p-Akt was still lower than that of the simple LPS group [48]. Xu et al. found that, compared with the hypoxia group, the expression of TNF-a, IL-6, and IL-1*β* mRNA in the group treated with resveratrol was significantly reduced compared with the hypoxia group. After resveratrol treatment or using LY294002 to inhibit PI3K, the expression of p-Akt decreased significantly. This shows that resveratrol can protect pulmonary artery smooth muscle cells by inhibiting the PI3K/Akt signaling pathway [49]. However, studies have also shown that resveratrol can increase the protein expression of p-Akt in vascular smooth muscle cells while inhibiting the expression of inflammatory factors, that is, by activating the PI3K/Akt signaling pathway to protect vascular smooth muscle [50]. Zong et al. found that when LPS acts on RAW 264.7 cells, the protein expression of p-Akt increases and the PI3K/Akt signaling pathway is activated. Under the combined action of resveratrol and LPS, the activation of the PI3K/Akt signaling pathway was more significant, and the protein expression of p-Akt was further higher than that of the LPS group [51]. It shows that Veratrol may play an anti-LPS-induced inflammatory response in RAW 264.7 cells by activating the PI3K/Akt signaling pathway. The systematic pharmacology part of this study shows that resveratrol can regulate inflammation pathways such as PI3K/Akt signaling pathway, NK-KB signaling pathway, and TNF-*α* signaling pathway.

### 3.3. Pathological Changes

**Figure 7:**
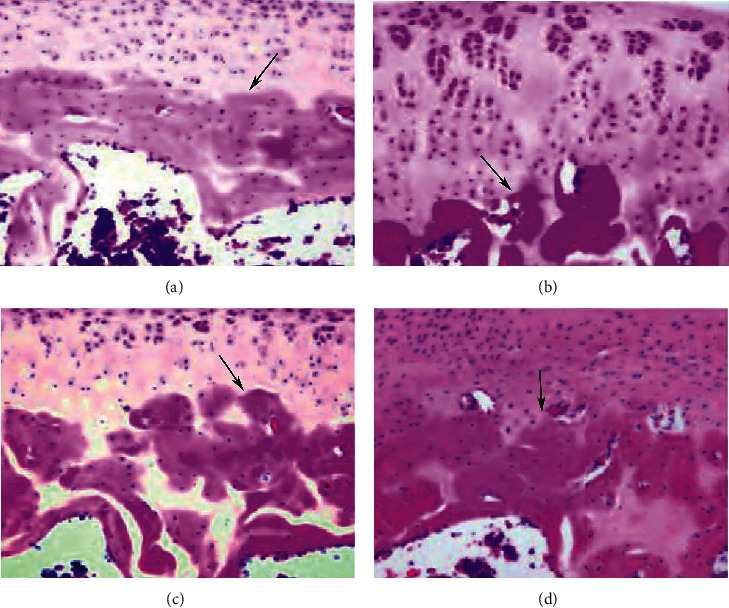
Pathological changes (HE staining; X200). (a) Control group; (b) model group; (c) resveratrol low-dose group; (d) resveratrol high-dose group. The black arrow indicates tidemark.

The surface of the articular cartilage of the blank control group is flat, the cartilage structure and tidemark of 4 layers are clear, and the chondrocytes are arranged neatly. The cartilage surface in the model group is irregular, cracks are generated, the tidemark recognition is poor, the cartilage structure of 4 layers is unclear, and the cartilage cells proliferate. The cartilage surface of the resveratrol low-dose and high-dose groups was more regular, cracks were reduced, a small amount of chondrocyte proliferation was seen, and the tidemark was visible. Compared with the model group, the degree of cartilage pathological damage was reduced (Figure 7).

### 3.4. Effect of Resveratrol on Serum IL-1*β*, IL-6, TNF-*α*, and MCP-1 Content

**Figure 8:**
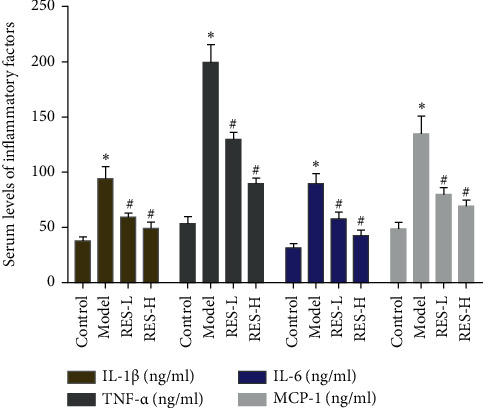
Effect of Resveratrol on Serum IL-1*β*, IL-6, TNF-*α*, MCP-1 Content (RES-L: resveratrol low-dose group; RES-H: resveratrol high-dose group; ^*∗*^compared with the control group, *P* < 0.05; ^#^compared with model group, *P* < 0.05).

Compared with the control group, the levels of IL-1*β*, IL-6, TNF-*α*, and MCP-1 in the model group were significantly increased (*P* < 0.05). Compared with the model group, the levels of IL-1*β*, IL-6, TNF-*α*, and MCP-1 in the resveratrol low-dose and high-dose groups were significantly reduced (*P* < 0.05) (Figure 8).

### 3.5. Effect of Resveratrol on the Expression of TLR-4, MyD88, and NF-Κb p65 Protein

**Figure 9:**
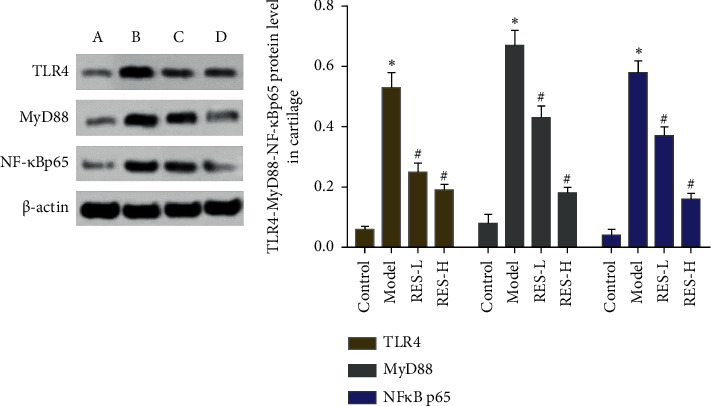
Effect of resveratrol on the expression of TLR-4, MyD88, NF-*κ*B p65 protein (A) control group; (B) model group; (C) resveratrol low-dose group; (D) resveratrol high-dose group. ^*∗*^Compared with the control group, *P* < 0.05; ^#^compared with model group, *P* < 0.05.

The expression levels of TLR-4, MyD88, and NF-*κ*B p65 protein in rat cartilage tissue were performed by Western blot. Compared with the control group, the expression of TLR-4, MyD88 and the ratio of NF-*κ*B p65 in the model group were significantly increased (*P* < 0.05). Compared with the model group, the expressions of TLR-4, MyD88, and NF-*κ*B p65 in the resveratrol low-dose and high-dose groups were significantly reduced (*P* < 0.05) (Figure 9).

### 3.6. Effect of Resveratrol on the Expression of Bcl-2, Bax, and Caspase-9 Protein

**Figure 10:**
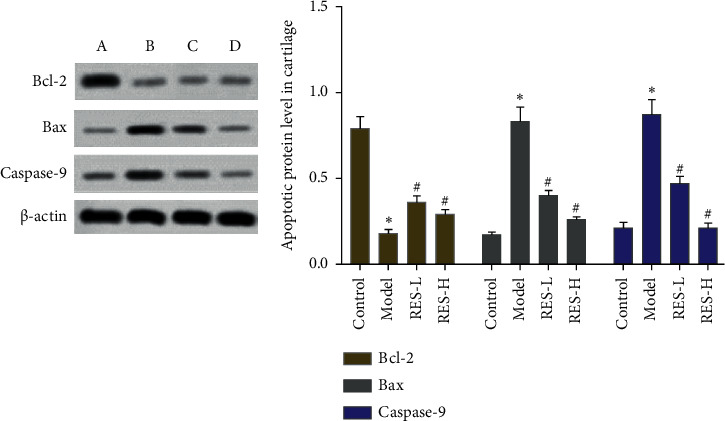
Effect of resveratrol on the expression of Bcl-2, Bax, Caspase-9 protein; (A) control group; (B) model group; (C) resveratrol low-dose group; (D) resveratrol high-dose group. ^*∗*^Compared with control group, *P* < 0.05; #compared with model group, *P* < 0.05.

Compared with the blank control group, the expression of Bcl-2 in the model group decreased (*P* < 0.05), and the expression of Bax and Caspase-9 increased (*P* < 0.05). Compared with the model group, the expression of Bcl-2 in the resveratrol low-dose and high-dose groups increased (*P* < 0.05), and the expression of Bax and Caspase-9 decreased (*P* < 0.05) (Figure 10).

Through network pharmacological analysis, this study found that resveratrol was concentrated in the TLR4 signaling pathway, and animal experiments were used to verify the results. Experimental studies have shown that resveratrol can improve the degree of pathological damage in OA rats. Apoptosis is positively correlated with the severity of cartilage destruction and matrix depletion in human osteoarthritis tissue specimens. The Caspase family plays an important role in the process of cell apoptosis, and the excessive activation of Caspase-9 and Caspase-3 in the apoptotic cascade can promote cell apoptosis. The antiapoptotic member Bcl2 can prevent cell apoptosis, while the proapoptotic member Bax is located in the outer mitochondrial membrane or cytoplasm and oligomerizes under stress to promote the release of factors from mitochondria, thereby triggering apoptosis [52]. Burlacu found that the ratio of Bcl-2 to Bax can reflect the relationship between Bcl-2 and Bax in the process of cell apoptosis: an increase in the ratio of Bcl2/Bax inhibits cell apoptosis, and vice versa promotes cell apoptosis [53]. In this experiment, resveratrol can reduce the level of Caspase-9 protein and increase the ratio of Bcl-2/Bax. It shows that resveratrol inhibits the apoptosis of chondrocytes in OA rats. In the pathology of bone and joint, proinflammatory cytokines play a key role. The levels of TNF-*α* and IL-6 are recognized cytokines that reflect the degree of inflammation. The high expression of IL-6 can lead to the degradation of articular cartilage and induce pain. TNF-*α* is an inflammatory response mediator secreted by monocytes and macrophages. Long-term high TNF-*α* levels may be the main reason for the development of OA in wounded joints. IL-17 is a proinflammatory cytokine secreted by T lymphocytes and monocytes. MCP-1 is a representative of the *β* subfamily of chemotactic cytokines and an important inflammatory response mediator of OA [54]. This study found that resveratrol can reduce the levels of IL-1*β*, IL-6, IL17, TNF-*α*, and MCP-1 in the serum of OA rats. It shows that resveratrol can maintain the normal structure and function of joints by reducing the content of proinflammatory cytokines.

TLR4/myeloid differentiation factor 88 (My D88) signal transduction pathway is an inflammatory signal pathway that has been studied in the prevention and treatment of knee OA in recent years [55]. TLR4 is an important signal pathway transduction protein, which is closely related to the pathogenesis of OA. It is highly expressed in OA chondrocytes and participates in cartilage destruction. It can activate various inflammatory factors through its downstream MyD88-dependent signal transduction pathway [56]. NF-*κ*B is a primary transcription factor that controls the expression of many proinflammatory genes and plays an important role in cell processes. Literature reports that plant extracts can affect the NF-*κ*B pathway [57]. Studies have reported that the use of siRNA to interfere with the expression of NF*κ*B p65 can reduce the pathological process of OA in rats, indicating that inhibiting the activity of NF-*κ*B can be used as a target for the treatment of osteoarthritis [58]. This study found that resveratrol downregulated the protein levels of TLR-4, MyD88 and the ratio of pNF-*κ*B p65/NF-*κ*B p65 in osteoarthritis rats. This shows that resveratrol can treat OA rats by inhibiting TLR-4/MyD88 and NF-*κ*B signaling pathways.

In summary, resveratrol improves the degree of pathological damage in rats with bone OA, reduces cartilage tissue apoptosis, increases the proportion of trabecular bone and the proportion of cartilage, inhibits the degradation of extracellular matrix, promotes the synthesis of extracellular matrix, and reduces the content of proinflammatory cytokines. This may be achieved through the NF-*κ*B signaling pathway mediated by TLR4. This study provides a theoretical basis for the clinical application of resveratrol in the treatment of OA.

## 4. Conclusion

Resveratrol may inhibit the activation of the TLR4-mediated NF-*κ*B signaling pathway and has a repairing effect on soft tissue damage in OA.

## Data Availability

The data that support the findings of this study are available in supplementary materials.

## Supplementary Materials

Supplementary Materials Table S1: Resveratrol Potential Targets. Table S2: OA genes. Table S3: Enrichment Analysis of Resveratrol-OA PPI Network.
