# Supplementary material for: Exploring the Mechanism of Resveratrol in Reducing the Soft Tissue Damage of Osteoarthritis Based on Network Pharmacology and Experimental Pharmacology
Source: Evid Based Complement Alternat Med. 2021 Oct 4;2021:9931957. doi: 10.1155/2021/9931957 (PMC8505078; doi:10.1155/2021/9931957)
Supplement: Supplementary Materials — Table S1: Resveratrol Potential Targets. Table S2: OA genes. Table S3: Enrichment Analysis of Resveratrol-OA PPI Network. [file 9931957.f1.zip › 9931957.f1/Table S1-revised (1).pdf]

Table S1 Resveratrol Potential Targets and OA genes

Resveratrol Potential Target set	BMP2
	STS
	ALB
	AKR1C2
	APOA2
	KIF11
	PDPK1
	MMP3
	PIK3CG
	NR3C2
	MAPK14
	PPP5C
	ADAM17
	ESR1
	GC
	MAPK10
	MAPK1
	CA2
	FNTA
	F2
	BCHE
	SRC
	CES1
	DPP4
	PIM1
	PDE4B
	DUSP6
	CTNNA1
	MMP8
	METAP2
	AKR1B1
	FGFR1
	CFD
	BACE1
	PDE4D
	AR
	MAOB
	SULT2A1
	PGF
	HSD11B1
	PTPN11
	RORA
	AKR1C1
	TTPA
	AKR1C3
	HSD17B1
	TTR

PPARD
NQO1
SEC14L2
WAS
GSK3B
PCK1
ADH5
CDK2
MMP13
PRKACA
ESR2
EGFR
ADH1C
CHEK1
GSTP1
GSTA1
MDM2
PPARA
SHBG
THRB
ADK
KDR
PTPN1
TGFBR1
TRAPPC3
NR1H3
ELANE
PGR
CYP2C9
SHMT1
HPGDS
NR1H2
PDK2
MET
LCK
DHODH
DCK
CCNA2
JAK3
F10
PRKCQ
ZAP70
MTAP
RXRA
MMP2
HDAC8
SYK
FKBP1A

RBP4
FABP3
FABP6
ITK
HNF4G
SERPINA1
ESRRG
FABP7
PYGL
REN
SULT2B1
NR3C1
LSS
NR1I2
PADI4
SULT1E1
GCK
HSP90AA1
AKT2
CTSS
AURKA
MMP12
ERBB4
ITGAL
S100A9
PLA2G2A
PPARG
DPEP1
NR1H4
XIAP
RARG
MAPKAPK2
HMGCR
TGM3
F11
CRABP2
CYP2C8
FECH
RARA
BRAF
IGF1
MME
HNMT
IL2
TEK
MAP2K1
ABL1
THRA

VDR
KIT
RARB
CTSB
CTSK
LTA4H
ARG1
HSP90AB1
PIK3R1
RXRB
TGFB2
ABO
MMP9
ADAM33
BHMT
INSR
PPP1CC
CASP1
BIRC7
TPSB2
PCTP
DTYMK
PROCR
PNMT
FKBP3
AMY1A
AMY1B
AMY1C
NFE2L2
APP
CA3
NQO2
CA5B
CA6
CA5A
CYP1B1
PTGS1
CA14
CA7
CA13
CA4
PTGS2
MAOA
CA12
CA9
CA1
GFER
TRPA1

CYP1A2
CYP3A4
PIK3CB
SLC6A2
CYP2C19
PIK3CA
TUBB1
ALDH5A1
ABAT
RELA
AKR1B10
MIF
AHR
CISD1
ALOX5
ODC1
GLO1
MAPT
G6PC
CXCL12
ABCG2
CYP1A1
F3
TYR
MMP1
KDM1A
KEAAP1
ADNP
SIRT1
CYP19A1
TUBB3
ABCB1
CLK1
PTK2B
ESRRA
ESRRB
IGF1R
ALOX15
HSD17B2
DYRK1A
DYRK1B
LTB4R
ERN1
HSD17B14
HDAC2
ABCC1
CBR1
CYP11B1

CYP11B2
CYP17A1
CCNE1
VCP
PLAT
CAPN1
DRD2
DUSP3
BCL2L1
BCL2
RAF1
CALM1
NOS1
TYMS
MTOR
IGFBP3
NOS2
NOS3
ADCY5
WEE1
FLT3
LIMK1
NOX4
YWHAG
CDK5R1
CDK5
CBX1
LMNA
LRRC26
KCNMA1
KCNMB1
KCNMB2
KCNMB4
MPHOSPH8
ARSA
PRKN
HDAC9
CASP7
CASP3
HTT
RORC
ATAD5
NFKB1
NFKB2
RAB9A
PTH1R
ACHE
GLS

TDP1
NPC1
TP53
ATXN2
SMN2
GLI3
HPGD
HIF1A
SMAD2
SMAD3
STK33
MEN1
KMT2A
BRCA1
POLK
PAK1
RGS4
CYP2D6
NR1I3
ALOX15B
RAPGEF4
PPM1D
NPR2
ALDH1A1
GCKR
vif
APOBEC3G
FH
BCR
GLP1R
GMNN
IDH1
GAA
MBNL1
PMP22
POLB
POLI
BLM
MYC
TSHR
TERT
CD38
CTSD
SIRT2
SIRT3
PRKCA
DUSP1
S1PR3

CDC25B
NFKBIA
FPR1
FPR2
S1PR1
BAP1
CTSL
NR5A1
ITGA4
C1S
ALPL
PTPN7
HTR1A
HTR1E
CTSG
HSPA8
ROCK2
NCOA1
MMP14
PTPN22
F12
RAC1
NCOA3
HRAS
NPY2R
EIF4G1
PRKD1
GALR2
STAT3
KLK5
RGS12
GNAI1
STAT1
HBB
BCL2L10
BCL2L2
PTGER2
BCL2A1
MCL1
NPY1R
HSPA1A
MPI
DLD
INS
NCF1
GNAO1
RAD51
RGS8

EIF2AK3
RGS7
RGS19
RGS16
TNFSF10
RUNX1
CBFB
ALOX12
SLC12A5
MCOLN3
JAK2
NPSR1
GLA
KCNH2
RBBP9
S1PR4
MAP3K3
MAP4K2
UBE2N
PHOSPHO1
NOD2
NOD1
PKM
KLF5
CTNNB1
OPRK1
PLEC
NPBWR1
GALK1
GSTO1
GPR55
EIF4H
UBA2
SAE1
UBE2I
PABPC1
GPR35
CACNA1B
NLRP1
GBA
PPME1
LYPLA1
TUBB
PPP1CA
MITF
NR2E3
PTBP1
OXTR

BCL2L11
GAPDH
APLNR
SENP8
ALPI
HHV8GK18_gp81
KCNQ1
SENP6
GSK3A
RXFP1
HDAC3
AVPR1A
PREPL
NLRP3
SLC2A3
ABCC2
FOXO3
AKT1
ALPG
IDE
SENP7
HCRTR1
CACNA1H
SLC6A3
PLA2G7
USP2
FURIN
MAP1LC3A
MDM4
TSG101
ATM
L3MBTL1
ADRB2
FEN1
EYA2
GRK2
SLC5A7
TRPV1
DRD1
APAF1
PAFAH1B2
PAFAH2
PAFAH1B3
TNNI3
TNNT2
TNNC1
APOBEC3A
TRHR

PLIN1
ABHD5
PLIN5
NTSR1
CTDSP1
CCR6
HKDC1
KAT2A
OPRD1
OPRM1
BAZ2B
DAGLB
CFTR
GOPC
ATG4B
KEAP1
PIN1
ARRB1
HTR5A
PRKAR2B
PRKACB
TLR9
NR0B1
CCT2
USP1
KPNB1
SNUPN
RAN
SMPD1
MC4R
FXN
P4HB
DNMT1
CRHR2
CRHBP
UCHL5
PSMD14
ABCB6
POLH
PTPN5
MRGPRX1
RIN1
HOXA9
CHRM1
CXCR6
FASN
TLR3
APOBEC3F

RAD54L
NR5A2
KCNK3
SUMO1
ARNT
TACC3
CHRM5
CHRM4
ID4
LARGE1
ERG
NSD2
RIPK2
GNAS
RACGAP1
EPAS1
TNFRSF10B
ASAP1
MCOLN1
env
TAAR1
PDE5A
PTAFR
TBXAS1
CYSLTR1
MC3R
MC5R
CHRM2
CHRM3
MAPK3
PPP3CA
FYN
ERBB2
PTPRC
HTR2A
ADORA1
ADORA2A
ADORA3
ADRA1D
ADRA2A
ADRA2B
ADRA2C
ADRB1
ADRB3
AGTR2
BDKRB2
CALCR
HTR2B

HTR2C
HTR6
SLC6A4
SIGMAR1
TACR1
TACR2
FLT1
VIPR1
CNR1
CCR2
CCR4
CCR5
CXCR1
CXCR2
CCKAR
CYP2A6
CYP2E1
DRD3
DRD4
EDNRA
HRH1
HRH2
EZH2
ACP1
GPR183
vpr
FGF22
RAD52
NAE1
UBE2M
MLLT3
PDIA6
RBBP8
GALR3
SIX1
TNF
CXCL8
WRN
TRA
MARVELD2
NCOA2
COPS5
SMARCA2
KLK7
TARDBP
PIP4K2A
SNCA
SIRT5

PAX8
S100A4
ERAP1
PRMT1
NR2F2
MBD2
SELE
CASP6
UHRF1
PLK1
ADAM10
PRNP
TDP2
PLCB3
PLCG1
RAPGEF3
FOXO1
PLA2G3
HSPB1
PSIP1
IL1B
NRP2
SLCO1B1
SLCO1B3
SIAE
tat
ATF6
JUN
PGD
GALC
CCDC88A
KCNJ6
CGAS
IFNG
YAP1
PI4K2A
GPR32
NPR1
SIRPA
CD47
FUS
NTMT1
ADCY9
IFNB1
HLA-B
SGK1
AICDA
GPR151

OA gene set	COL2A1
	ACAN
	SMAD3
	FRZB
	COMP
	GDF5
	MATN3
	MMP13
	COL11A2
	ASPN
	CCAL1
	IL1B
	COL9A1
	PTGS2
	TNF
	IL6
	MMP3
	TNFRSF11B
	TGFB1
	MMP1
	ADAMTS5
	COL11A1
	FBN1
	IL1RN
	UFSP2
	OS4
	OS6
	TGFBR1
	COL6A2
	COL1A1
	TGFBR2
	ADAMTS4
	IL17A
	CILP
	SLC26A2
	IL1A
	LOC109461476
	CXCL8
	COL9A2
	BGLAP
	C4orf47
	TRPV4
	TRAPPC2
	TNFSF11
	TGFB3
	TIMP1
	MMP9

CRP
BMP5
AEBP1
DDR2
PTGS1
SOX9
COL9A3
LRCH1
IL10
MGP
MATN1
CLCN7
CHI3L1
MCF2L
ADAM10
TNFAIP6
KIF22
HAPLN1
PRG4
ADAMTS14
COL10A1
CCN6
UCMA
COL5A1
XYLT1
UFC1
COL5A2
EBF3
MED13L
HOTAIR
H19
PACERR
ANKH
CANT1
MEG3
KCNK15-AS1
COL1A2
TMSB4X
ESR1
GAS5
TGFB2
MIR140
VDR
HFE
COL3A1
LEP
LOXL3
LOC102723692

HLA-B
VEGFA
PCGEM1
PHEX
MIR146A
MEFV
PMS2P2
KCNJ5
KCTD13
IL1R1
ADIPOQ
MMP2
TLR4
IGF1
EFEMP2
IL18
SMAD2
CCL2
ALB
SPP1
RUNX2
DDH2
F9
CD36
CASR
PTPN22
GHR
BMP2
LOC101448202
IL4
HGD
FGFR3
DCN
MIR155
SMAD6
TNFRSF1A
NOS2
GNAS
GBA
PTHLH
ACP5
IRF5
LRP5
CALCA
ACTA2
MYH11
CXCL12
CCL5

MYLK
FMOD
ADAM12
HMGB1
IL13
PPARG
IFNG
SLC2A10
IBSP
HPGD
LOC101928222
TIMP2
F8
TRPS1
TLR2
ATP7B
HLA-DRB1
BMP7
CTSK
AKR1C1
ICAM1
EXT1
RELN
CD44
IL15
SIRT1
CCN2
MMP8
LMX1B
SYK
TNFRSF1B
SOST
PLAU
FAS
ENPP1
PLOD1
TCIRG1
IL1RAPL2
WWP2
CENPP
COG5
HIF1A
IL7
TNFRSF11A
ACE
UFL1
EXT2
SCARB2

SERPINH1
PTH
VCAM1
AP3D1
BMP6
ADAMTSL1
SLC22A4
IHH
FGF2
CCL3
MAPK14
FN1
JUN
SPARC
LMNA
RETN
OTULIN
PHEX-AS1
PTCHD1-AS
LOC100130744
LOC106627981
HLA-DQB1
CSF1
IL2
CXCL1
BTNL2
OSM
EFEMP1
TYR
CIITA
KL
ESR2
TP53
SOD1
IGFBP3
OPTC
ADAMTS3
NLRP3
IL18R1
AIP
PLA2G4A
MIR150
FTO
MAPK1
FKBP14
LOC113939944
CCR6
GNL3

MAPK8
FOS
DKK1
COL6A4P1
SOD2
MEN1
NAMPT
SERPINE1
IL17F
CTSB
SLC40A1
ZMPSTE24
KIF7
GPR101
ZNF687
IL11
CRH
NOTCH1
CALM1
PTGES
DOT1L
LRP6
GNA11
PSAP
PLAUR
TLR5
C1S
TGM2
GRIN2B
SAA1
OPRM1
ALOX5
TRPV1
TLR8
ELN
IL4R
LTBP1
ADAM17
THBS1
POMC
CPT2
CD244
COG4
NFKBIL1
MIR132
SPDT
BMP1
F2RL1

BCL2
CXCR4
FGFR1
ENG
CTNNB1
CASP3
TLR8-AS1
S100A12
S100A9
FASLG
NFKB1
UQCC1
SP7
IL23A
TNC
LEPR
RHOB
CCL20
FSTL1
HTRA1
HSPA5
CAT
SERPINF1
HLA-DQA2
TGFA
NGF
RELA
BMP4
MMP14
WNT1
PTH1R
CCR5
ELANE
PEPD
MIR181A1
MMP7
NFKBIA
DIO2
MPO
HP
CCN4
SOX5
AGER
ALDH1A2
ADAMTS9
MIR34A
ACVRL1
RET

BRCA2
IDS
LYZ
ABCC9
ARID1B
LTBP2
TMEM38B
SLCO2A1
CREB3L1
FKBP10
P3H1
FUNDC2
FKBP14-AS1
LOC106050102
HGF
EPAS1
COMT
GLT8D1
MIF
LTA
SUPT3H
CX3CL1
DSPP
NCOA3
MTHFR
HSPD1
TTR
IL16
FOXP3
IGF2
CSF2
COX5A
IL6R
TIMP3
BAX
SERPINA3
IL5
BDNF
UBA5
UFM1
CDK5RAP3
DDRGK1
UFSP1
CASP1
VIP
FBLN2
NLRP1
DUS4L

FGF18
HMOX1
B2M
GREM1
PLA2G2A
CTSL
DMP1
PON1
PITX1
NEAT1
MRC1
GPR22
ANXA1
ITGB1
HLA-DQA1
CP
ADAMTS1
LPAR1
IL1RL2
MIR149
FGF23
CXCR3
NFE2L2
PTN
MIA2
CXCL10
IFRD1
MMP17
LEPQTL1
GPT
IL17RA
IFNB1
LOX
NCOR2
PARD3B
CTSD
PBRM1
CDC5L
S100A8
IL32
COL27A1
LIF
P4HB
CANX
MIR26A1
MIR9-1
VIM
CCL18

STAT3
BDKRB2
CHADL
IL2RA
TRAF6
EGF
IL9
HAS1
FOXO3
CLEC3B
CCR2
HRH2
NME8
VIM2P
CASP8
GSR
CCL4
MIR145
TLR10
DANCR
TGIF1
VCAN
PARP1
CXCL13
SCN9A
PAPSS2
NOX4
CYP27B1
HDAC4
MMP12
TRAF1
HSP90B1
PIK3CG
PADI4
LRRK2
IDUA
APOA1
CALCR
SOCS1
NR3C1
PTK2B
CCR1
PROCR
CSF1R
CD40
NPY
FOXO1
TLR9

SERPINA1
HDAC9
ERG
FGF1
MCAM
TRAPPC2B
HAS2
POSTN
IGFBP7
MIR130A
CD68
FOSL1
NOD2
MEPE
CCND1
MIR29A
MIR29B1
GLIS3
YAP1
LGALS3
IL37
DNMT3A
DEAF1
PRKCA
ITGB3
KNG1
CDH2
ATF2
GAPDH
TNFSF13B
GJA1
CCR3
IL1R2
HAVCR2
MAZ
XIST
FBLN1
TNR
TNFAIP3
TLR3
CXCR5
TIMP4
MIR335
SELL
STAT4
ALPL
LTBP3
ASTN2

CTLA4
WNT3A
SST
IGFBP5
EDN1
SP1
CLU
TACR1
MALAT1
PLOD2
TPI1
MTOR
ANP32A
MIR223
CAMK2B
TGFBR3
SLC39A8
MUS81
XRCC4
CRTC1
PLEC
CSMD1
SPCS1
DNAH10
NACA2
RWDD2B
TMEM167A
SCARNA18
TSBP1-AS1
FLT1
THBS3
TFEB
AGTR1
GALNS
SYVN1
TRAPPC3
TRAPPC4
TRAPPC10
TRAPPC9
TRAPPC12
TRAPPC6A
TRAPPC2L
TRAPPC1
TRAPPC5
TRAPPC8
TECTB
TRAPPC3L
INHBA

LUM
MMP28
MIR210
AR
PLAT
RBFOX1
CILP2
LGALS1
OLR1
CREB1
DIO3
CCR7
SFRP4
CDKN1A
CYP19A1
PTGER4
CA2
SIRT6
LY96
MC1R
MIR671
SLBP
THRA
USP33
MIR98
CXADR
IL21
UCN
CXCL6
MIR27A
MIR4435-2
SELE
SLC17A5
WNT16
DCSTAMP
TENT5A
TRPV6
XDH
RARRES2
PDIA4
RAC1
CXCL5
MIR195
ANGPT1
MET
ALOX15
JUND
WNT5A

ANXA5
F5
HLA-DPB1
PRKAR2B
BCAP29
HPBP
FGFR2
SYNE3
GNAS-AS1
ADRB2
OCRL
TSPO
SOX6
PREP
FOLR2
ZFAS1
IL1RAP
IL1RL1
MIR15A
IL34
MIR21
PADI2
SLC11A1
SNRPB
CD86
TECTA
CEACAM16
OTOG
TH
PECAM1
IGF1R
ANPEP
CD14
SERPINC1
GHRL
SAA4
OPRD1
CXCR1
LCN2
IL18RAP
AQP1
SRC
HLA-A
GLB1
SIRT2
CHST11
SHOX
MYC

PRDX5
ITGB5
EDEM2
LINC00976
MMP10
SLC34A1
TWIST1
FAP
STC1
OLIG3
MIR9-3
MIR9-2
CAV1
AKT1
CXCR2
MAP2K4
ITGB2
CDKN2B-AS1
PNOC
CD40LG
MITF
CHI3L2
ADIPOR1
PDPN
PIK3R1
CD247
ADCYAP1
TREH
AKNA
PART1
MIR381
IL22
JAG1
F3
IL33
BMPR1A
SMO
BSG
TNFSF13
ACTG1
NR1D1
HDAC7
TYMP
CALM2
CRYAB
NTN1
CCL11
SERPINB2

SREBF2
CRLF1
ITLN1
FFAR4
UNC5B
SPON1
MIR141
MIR16-1
MIR204
MIR26B
MIR22
MIR27B
MIR33B
MIR488
FOXD2-AS1
ADAMTS8
SNORD19
CXCL2
ENO1
P4HA2
DPP4
PGF
CCL21
ROR2
GUSB
IL24
SLPI
CEMIP
MIR193B
MIR365A
GPI
OLAH
ADAMTS15
HMMR
NUCB2
BIRC5
GZMA
TCF7L1
F2
GHRHR
FLNA
GPT2
SLC26A4
FBN2
ANKAR
H2AC18
NEMP2
PDCD5

CX3CR1
TFRC
CRYAA
FAM20C
ALMS1
SLC34A3
GBP4
SMAP2
PALM
PALMD
PTEN
ITGAM
IL10RA
B3GAT3
IL3
ADIPOR2
CCL8
CLEC4A
CYTL1
MIR320A
TPSAB1
MIRLET7E
MIR454
MIR885
BGN
ALPP
FKBP5
UGDH
TNXB
HPSE
IGFBP2
CARD16
CCN1
MIR221
PRELP
CHAD
CTSG
MYD88
CSGALNACT1
APLN
PRTN3
CSK
ATP6V0A2
TBX4
PLEKHM1
USP50
GSK3B
SPHK1

CNR1
CD38
HBEGF
SP3
KLF2
SOD3
PCSK6
LGALS9
MIR199A1
MIR199A2
MIR675
CD163
PRL
ITGAL
SDC1
SMURF2
MIR320C1
NOS1
CHRDL2
PAK1
TUG1
NLRP12
HRH4
SEMA3A
RPA3
CXCL11
HBP1
IL36A
MIR125B2
MIR125B1
LOC111365141
NOG
IL23R
DUSP1
NGFR
S100B
MMP16
ARNTL
CCL22
MIR455
SDC4
LNCRNA-ATB
CYCS
SELP
TEK
GGT1
KDM1A
CD80

STEAP4
TNFRSF10A
JAM3
STAT1
CFI
CSF3
MAP2K6
ATF3
MIR30C1
C3
GRN
DDR1
PPARGC1A
CAST
MAF
HMGA2
B4GALT1
CST3
GPD1L
CD47
AHSG
HYAL2
SFRP1
LGALS8
CABIN1
HMGB2
MRC2
ADAMTS7
CIRBP
MIR582
MIR590
MSH6
TMPO
CNR2
BAG6
ATF6
KCNJ11
HMGCR
XBP1
GLRX
MUTYH
ORAI1
UTS2
COL6A4P2
GPR33
IL18BP
THBD
RECK

NT5E
VEGFC
ALK
CDKN2A
NOS3
ETS1
LEF1
MAP3K5
CYBA
OPRK1
SHC1
CCR4
KDM6B
PTGES3
PPBP
SERPINB5
SERPINE2
TFF3
SERPINA4
PITRM1
IL27
CCL13
IFNL1
MIR181A2
MIR30A
MIR206
MIR139
MIR101-1
ERVK-18
POR
PTGER2
CYP1A1
TCF4
ADM
NCALD
HHIP
NRCAM
SIK3
LRG1
MIR4435-2HG
MELTF-AS1
NOD1
AQP9
MAP1LC3A
ETS2
STAB1
SCUBE1
IGFBP1

PRKN
PDCD1
PPARA
KIR2DS4
INS
DSP
CSTA
TGIF2
FOXD1
TLR1
FAH
HPD
PSTPIP1
FMR1
CHST14
HPS6
MRAP
SAA2
AHR
YY1
GALE
HABP2
KLRB1
HIF3A
FIP1L1
PRNCR1
IL2RB
ADA
PLA2G3
EZH2
NR1H3
XYLT2
IL12A
C5AR1
GSTM1
SLC23A2
BBC3
FGF21
SCG2
GSTT1
MIR29B2
MIR29C
MIR483
MIR338
NKILA
CALR
HSD11B1
LRP1

SMAD4
APOH
L3MBTL2
L13304-024
AB372731
HSALNG0135621
TNFRSF6B
TMED2
ATM
BMPR2
CSF2RA
CTSS
BRD4
KPNA2
CS
MSTN
ULK1
PTH2R
SFTPB
SFTPC
SFTPD
DNTT
SFTPA1
CMKLR1
MFAP4
SLC25A27
MIR23A
HOTAIRM1
MIR186
MIR940
MIR300
OAP
LOC106728418
CHIT1
TNIP1
THY1
PYCARD
IL19
IL22RA1
EP300
KAT5
ITIH1
ILF3
CEACAM4
CHUK
CCK
TREM1
CXCL9

ADAM15
SMPD1
PPP3CA
GSTP1
ESRRB
EDNRA
PIK3CB
PDPK1
CYP2D6
TP63
KEAP1
ENO2
FAAH
CD3D
CSNK1E
TRAF3
SOX2
PRKAA1
C5
HCFC1
HYAL1
BECN1
DVL2
NCAM1
TARDBP
RIPK2
DLL1
APOB
PKM
SHMT2
MECP2
FOXM1
BCL2L11
KLF5
MFGE8
GANAB
HLA-G
NFATC4
ACSL4
BRD2
RBP4
WIF1
TRAF2
TFF1
SPHK2
SULT2A1
PDE5A
PFKFB3

PDE4A
NR1I3
PTPRD
COL6A3
LOXL2
HES1
GPX3
LGR5
CSPG4
DDIT4
PTGES2
TRIM24
ZFP36L1
TAGLN
STT3B
PRDX4
PDGFC
RSPO2
RIPK4
APOL1
KHDRBS1
CBX4
BAG1
EIF4G2
VPS4B
RIPK3
SECISBP2
LPA
DGCR8
CHKA
CCL7
UBE2M
SOX11
SMN1
NOX1
FUT1
DPP7
IL17RC
HS6ST2
HGFAC
CPEB1
DKK3
ABCD2
UNC5C
TFF2
SDCBP
KLK11
CD160

DSTN
BOK
TSC22D3
TUFT1
OXA1L
UHRF1
LGR4
FOXP4
CREB3
BCL2L13
EN1
FIS1
DUSP19
SUB1
CCL16
CRTAC1
RACK1
NID2
FUT4
LAG3
CCAR2
CKLF
NKX3-2
YAF2
CORT
CCL14
CCL15
TRIM59
ATG2B
HSH2D
GRINA
MICAL3
FNDC5
TM2D3
SHROOM1
XCL2
TUT7
KCNQ1OT1
CASC2
PVT1
MIR196A2
MIR138-2
MIR152
MIR200B
MIR30B
MIR370
MIR373
MIR181C

MIR449A
SNHG1
MIR93
MIR128-1
MIR138-1
MIR375
MIR107
MIR23B
MIR301A
MIR211
MIR24-1
SNHG15
SNHG5
MIR124-3
MIR16-2
MIR193A
MIR451A
MIR129-1
MIR129-2
MIR324
MIR337
MIR106A
MIR24-2
MIR377
SNHG16
LOXL1-AS1
MIR361
MIR486-1
MIR577
MIR411
SNHG14
MIR1236
MIR105-1
MIR1277
LINC00662
MIR634
MIR1202
MIR1246
TP53COR1
LOC112543491
LOC110283621
JAK2
APP
LPL
XIAP
C1R
ADAMTS2
CEACAM8

FCGR2B
HTR2A
MAP3K8
CR1
FGF7
SRF
LECT2
CEBPB
TNFSF14
HDAC1
CFLAR
CD28
IL6ST
MAPK10
HDAC2
PTPRC
RAG1
GAL
SNAI1
GNS
ALCAM
RAG2
SHBG
ATP6V0E2
SULF1
FGFR4
VWF
PRODH
NPC1
ERCC6
GALC
FMO1
GH1
PIGB
PDZD11
MAPT
USP8
TRIM32
ABT1
SLC2A1
TSLP
MATN4
MAP2K7
MYDGF
WNT7A
LDHB
SETD2
TNKS

SETD1A
P4HA1
CA10
HES5
MIR146B
BHD
ITGAV
MBL2
FCGR3A
GDF6
SIGLEC1
PRRC2A
EGR1
PDGFRA
PRKG1
ACTB
MAT2A
NR4A2
TYRP1
SLC26A3
REL
FBLN5
SLC26A5
MFAP5
SLC26A8
CCRL2
LTBP4
SLC26A1
SLC26A6
SLC26A11
FCRL3
CAPN14
GGT2
MACIR
GGTLC3
TMPO-AS1
TGFB2-AS1
TGFB2-OT1
ENSG00000259039
JUNB
IKBKE
STAT5B
LBR
FGA
AMPD2
FGF3
CD34
GGT3P

SOCS3
LRP2
S100A4
CD151
NTRK1
ALOX5AP
CD79A
BST1
FCGR3B
PCSK9
NF1
CYP11A1
NPM1
GPD2
SLC25A13
AP3B1
GAK
HERC2
ATP2B1
ADD1
CDC7
PAX7
SOCS2
GRK4
HTT
HK3
CRADD
EIF2B1
MMP24
USP24
TACC3
SLC39A4
ITIH4
LCT
NFAT5
EEF1D
CRBN
ANXA3
DPEP1
PUF60
SPTBN2
PTPRG
SLC30A10
SCYL1
MCAT
RASGRP3
SMYD3
JPH3

CYP2A13
SCRIB
AGAP1
MAGI1
MAT2B
HLA-DPA1
DYNC1I1
CEACAM3
ABCB9
EIF6
RNF4
STRA6
POLA2
PRDM2
SART1
SHARPIN
SLC44A2
PAPPA
SH3BP2
NISCH
NEK4
CPNE1
GALNT5
MAF1
FAR2
GGT7
GBX2
CPSF6
AAGAB
B9D2
BMP2K
BTN3A1
CEP250
NCAM2
RNASEH2C
RFX3
UNC5A
YKT6
TIPIN
SMG6
PLXNC1
SF3B2
RAD54L2
ZMYND8
KCNIP4
MAEA
KANSL1
ACER3

TCTN2
SF1
RPL29
RPGRIP1L
NCOA6
FOXL1
DDX10
IRX3
LRIG3
EXOSC4
EYA2
CDK2AP1
DLGAP2
BTN2A1
ANKRD6
CHD9
NDST4
SNX32
MRPL11
SENP6
PARP10
RFT1
HNRNPUL1
RBM39
TNIP2
FRMD4A
COLGALT2
ISLR
LRRN1
LYPLAL1
NELFA
FAM136A
ERGIC3
ABHD14B
BTN2A2
TAS1R2
RBM6
VPS51
TTI1
POGK
RPRD2
PPM1H
RAPH1
RBM12
TNFSF8
APOBEC4
FSTL5
ISLR2

H1-4
KIF12
KIAA1217
KIF26B
LMAN1L
DDX55
DHX35
ERP27
BTN3A2
ATF7IP
ANAPC4
AAR2
CCDC33
TMEM91
TTLL12
MOB3B
SBNO1
NSD2
RP1L1
SF3A2
MPHOSPH9
ZCCHC8
C11orf80
ACTR5
CCDC91
RALGAPB
ZWILCH
UVSSA
ZFYVE28
SRBD1
NT5DC2
FILIP1
BTN3A3
GSDMC
HAUS3
GALNT18
H4-16
HECW1
EPPK1
NRBP2
TSKU
ZFPL1
TENM2
RRP9
KNTC1
KLHL42
ZC3H3
IQCH

H1-2
IZUMO4
HMGN4
NKAIN1
BRINP1
BTNL8
EIF1AD
TSPAN17
SPATC1
THSD7B
MLXIP
RSRC2
KAZN
CCDC97
CEACAM21
ZBED5
POLR2M
H3C3
LURAP1L
LRRC37A
FAM53A
FAM83C
FAM193A
FAM98A
C12orf60
KIAA1755
H1-3
H2BC4
H2BC9
LSMEM1
CLEC18A
TMEM129
TMEM241
EQTN
CNBD2
CLEC18C
ZNRD2
PLPP6
C11orf87
H2AC6
ATP23
ZNF345
TMEM61
C8orf82
ATP6AP1L
C16orf95
CCDC26
SMIM4

MS4A13
PCNX3
MANSC4
RFLNA
STIMATE
MINAR1
DELEC1
BTN2A3P
SMIM23
LINC01006
DNAH10OS
LINC00114
MIR661
SNHG17
GDF5-AS1
HCG11
PDXDC2P
GUSBP2
CTBP1-DT
NOP14-AS1
LINC01088
LINC02210
LINC00977
SNORD16
SAMMSON
IQCH-AS1
MIR4286
HCG23
FAM83C-AS1
NR2F2-AS1
SNORD19B
ILF3-DT
LINC01094
LINC02210-CRHR1
LRRC37A4P
LOC100129917
LINC01507
ZC3H11B
ASTN2-AS1
MIR1289-1
GARS1-DT
ATP2B1-AS1
DUBR
LOC102546299
MMP24OS
LOC157273
LOC349160
LYPLAL1-AS1

NSFP1
MDFIC2
LINC01574
DDX19A-DT
DUS4L-BCAP29
ENSG00000255224
LOC728975
ROCR
LSP1P3
HOMER3-AS1
LINC02399
KIF26B-AS1
LOC101928731
LOC101929054
TMED2-DT
ZNF664-RFLNA
SBNO1-AS1
LINC01754
LINC01338
LINC02234
LOC101928540
ENSG00000254632
ENSG00000272462
ENSG00000253926
ENSG00000255730
ENSG00000261324
ENSG00000261384
ENSG00000254855
ENSG00000254461
ENSG00000255038
ENSG00000255320
ENSG00000237174
ENSG00000206249
LOC729254
SLBP-DT
RPL35AP
ENSG00000245156
LINC02742
LINC02650
MAPK8IP1P1
MAPK8IP1P2
MTND4P14
RNA5SP301
ENSG00000252443
ENSG00000254094
ENSG00000261578
ENSG00000249664
ENSG00000258216

ENSG00000260233
ENSG00000231612
ENSG00000234091
ENSG00000228322
ENSG00000227554
NPM1P14
RN7SKP173
LINC02752
H2AC5P
LINC02680
RNA5SP349
RNA5SP355
ENSG00000257042
ENSG00000275443
ENSG00000282246
ENSG00000250098
ENSG00000260773
ENSG00000250453
ENSG00000234892
ENSG00000223727
ENSG00000200998
ENSG00000237530
ENSG00000238224
LOC102724929
SOD2P1
TPT1P9
MPPED2-AS1
PPIAP1
RPL26P20
ENSG00000248112
CNN2P10
LOC101928268
ENSG00000272588
ENSG00000273771
ENSG00000274874
ENSG00000285108
ENSG00000251446
ENSG00000279093
ENSG00000285668
ENSG00000287373
ENSG00000273179
ENSG00000238232
ENSG00000173727
ENSG00000207009
ENSG00000238156
NPIPB14P
NPAP1P4
RPL21P34

MRPS33P3
HSALNG0046573
lnc-GLIS3-2
lnc-KCNK7-4
lnc-SSSCA1-1
lnc-IQCH-7
MARK2P15
LOC105369212
ENSG00000283517
ENSG00000285914
ENSG00000286910
ENSG00000285577
ENSG00000280904
ENSG00000285144
ENSG00000277749
ENSG00000219682
lnc-GGCT-3
lnc-PLEC-4
HSALNG0026167
HSALNG0032399
HSALNG0032401
ENSG00000239793
ENSG00000287632
ENSG00000288096
ENSG00000288639
lnc-ZMYND8-10
HSALNG0005615
FJ601676
HSALNG0048733
HSALNG0035397
HSALNG0026174
NONHSAG001562.2
piR-40096
piR-43098
HSALNG0069708
HSALNG0069709
HSALNG0069710
HSALNG0123133
lnc-ADD1-6
lnc-APOBEC4-5
NONHSAG011935.2
lnc-FAM83C-3
lnc-PRPF18-2
lnc-SOCS2-6
lnc-STAB1-1
lnc-TIMM29-1
lnc-CTDSP2-7
lnc-FAM83C-2

lnc-TTLL12-1
lnc-YKT6-2
MK280269-012
piR-31751
piR-34822-068
RF00017-6801
RF00017-6803
SNODB718
HSALNG0094715
L13304-005
lnc-ADD2-2
lnc-KIF12-2
lnc-KIF12-3
piR-39858-250
piR-50208
piR-50446
RF00017-2167
RF00017-2535
RF00017-4662
H3P27
ENSG00000256378
ENSG00000280309
ENSG00000277135
ENSG00000286417
LOC107986989
lnc-NT5DC2-1
lnc-PBRM1-2
lnc-TACC3-3
HSALNG0046567-001
HSALNG0059706
SMC4P1
RPL14P6
5MWI_A-023
HSALNG0023242
HSALNG0059707
HSALNG0032308
HSALNG0026168
HSALNG0026169
HSALNG0026175
HSALNG0026176
HSALNG0050296
HSALNG0092966
HSALNG0123135
HSALNG0126180
HSALNG0126183
HSALNG0092958-001
HSALNG0111532
HSALNG0130549-001

HSALNG0130549-002
lnc-ADAM10-10
piR-36756-038
piR-43107-151
RF00017-2855
HSALNG0123949
MN308750
HSALNG0073879
HSALNG0093138
HSALNG0094803
HSALNG0106203
HSALNG0127649
HSALNG0129967
lnc-HFM1-3
lnc-KLHL42-2
lnc-NEK4-1
LOC101929770
ENSG00000274075
ENSG00000286953
ENSG00000279304
ENSG00000280022
ENSG00000286018
ENSG00000278892
HSALNG0010676
HSALNG0010680-001
HSALNG0012153-001
LOC105369890
LOC105376839
LOC107986000
lnc-COLGALT2-6
lnc-FGF18-4
HSALNG0057639
HSALNG0015811
HSALNG0063314
MF281437-018
ENSG00000244712
HSALNG0012153-002
HSALNG0046568
HSALNG0032304
HSALNG0032306
HSALNG0033405
HSALNG0043117
HSALNG0043118
HSALNG0046882
HSALNG0050314
HSALNG0117900
HSALNG0117901
NONHSAG012622.2

HSALNG0073878
HSALNG0112393
HSALNG0113623
HSALNG0124661
piR-49204-003
lnc-LINC02210-CRHR1-1
piR-56497-029
piR-57133-366
piR-61945-087
RF00004-010
RF00017-1212
RF00017-4614
RF00100-018
HSALNG0085882
lnc-ARHGDIB-2
lnc-LYPLAL1-11
lnc-TMEM167A-7
lnc-TNC-7
piR-46368
RF00994-385
HSALNG0076293
HSALNG0090065
HSALNG0107171
HSALNG0133078
piR-30534
piR-32023-046
piR-52471-007
piR-60429
RF00001-070
LOC105377721
LOC107986600
LOC107985895
HSALNG0043116
HSALNG0046883-002
HSALNG0116887
piR-56497-036
RF00017-2174
HSALNG0116918
piR-49732-244
piR-57133-262
KDR
EPHB4
PRKCZ
CAPN2
NRP1
ANG
GC
CYP2C9

WRN
IKBKB
TNFRSF10B
S100A11
ANGPTL2
BMP3
PRKAR1A
ACVR1
CDH1
PSMB8
SNCA
GLA
ZEB1
IKZF1
F7
CYP24A1
CDKN1B
ZEB2
PINK1
ASAH1
ARSA
PARK7
GALNT3
PLOD3
COL4A3
LAMP2
COL4A4
M6PR
ATP13A2
LOXL1
UGCG
SLC39A13
PECR
GSTM5
NPC2
GBA2
COLEC12
BLZF1
ICOSLG
LZTS1
COL15A1
SYNJ2BP
PLEKHA7
STARD10
PCMTD1
EMP1
COL6A5
EMSY

PHYHD1
MIR10B
MIR222
MIR212
MIR103A1
MIR664A
MIR6069
ATG7
MIR136
KLF3-AS1
MAP3K7
ITGA4
CD55
ICAM3
ELF3
DEFB4A
MIR92A1
CXCL16
EFNB2
TNFSF12
DEFB103B
PF4
ARL13B
FCGR2A
HLA-DRB5
LTB
FLG
GPBAR1
BTLA
CPOX
ANXA6
ZFP36
MAP2K3
ANXA2
TNFSF10
MAP3K2
VTN
SMAD7
AIF1
F2R
TAC1
HNF4A
IRAK3
LMNB1
CPB2
COL4A5
ESD
SERPINF2

CHIA
DSE
NPPC
LAMP3
DNMT1
FST
FURIN
HSD11B2
AMBP
E2F2
GADD45B
OSCAR
POLG
LTF
TIA1
ELAVL1
HOMER1
CD58
IL20
ZBTB38
MIR203A
FAM215A
MIR448
FZD1
ERBB2
PDGFB
PGK1
CA1
CD59
DDIT3
CFP
PLA2G5
LGALS3BP
POMGNT2
PROC
HDC
ITGA1
SMAD5
PTX3
HAS3
SOX4
TNFRSF13C
LAIR1
RNASE3
GRIN2D
GNAQ
RIPK1
GK

HLA-DRA
GNAI3
SLC6A4
SH2D1A
HTR3A
ATIC
SLC19A1
PAX9
PPP1CB
LFNG
DHODH
SLC9A3R1
RELB
PPP1CC
SERPIND1
HTR3B
MCOLN1
CACNA1F
STS
MAFB
CLCN5
CLCN4
PAX1
ROBO3
GPR143
CHST3
MEOX1
SUCLG2
OCA2
RPS26
MICB
HLA-DMB
BAZ1B
CD69
HLA-DOA
HLA-DMA
CARD8
GDF3
GNPTAB
ZBTB20
SLC24A5
NPSR1
COPE
GPRC6A
HPS4
HPS5
GNPTG
DDX39B

TBX6
SLC35D1
SLC45A2
MLANA
FRMD7
MICA
MYO18B
CABP4
TBL1X
TAGAP
RIPPLY2
PACSIN3
COG1
HES7
CLEC16A
SUPT20H
TGDS
PLB1
SIAE
ZNF334
MESP2
YIPF3
ZNF664
LACC1
SHROOM2
LRMDA
TMSB15A
RIPPLY1
KCNJ18
UCA1
SNHG29
FBXL19-AS1
SNHG28
AP4B1-AS1
LURAP1L-AS1
MIR4497
MYO18B-AS1
GPR143P
OASD
OA3
TERT
ZAP70
BMPR1B
MAPK3
GSN
MAPK9
TAB2
RHOA

FPR2
IL7R
IRF1
FHL2
SLC9A3
ANGPT2
CHRNA7
TRPA1
LTB4R
ICAM2
TICAM1
S1PR1
P2RX1
TRIB3
NRP2
SIRPA
TNFRSF17
CD209
LTB4R2
IGFBP4
CHGA
FCGR1A
LTBR
IL17B
BPI
ACKR3
CAMP
CCL19
SEMA3F
KIR3DL1
CTHRC1
KCNIP3
KIR2DL4
ADGRE5
KIR3DL2
ADGRE2
XCL1
SPA17
GALNT15
KIR2DL1
KIR2DL3
KIR3DL3
VASH1
ZC3H12A
PCOLCE2
SNX19
LVRN
FNDC1

SAAL1
KIR2DL2
KIR2DS2
KIR2DS3
KIR2DL5A
KIR2DS1
KIR3DS1
MIR17
MIR127
MIR31
MIR33A
MIR148A
KIR2DP1
HDAC8
TXN
RAMP2
CXCR6
ADAMTS12
MIR95
NFKB2
APOE
GRM1
DBH
ROCK1
KCNQ1
F10
CD4
PRKDC
PDE3A
MVK
PBX1
PPIB
ADRB1
COL4A1
ASS1
CBS
PAH
PDE4D
ADSL
GAA
IKBKG
LIPA
NFATC1
GHSR
AK1
ABCC2
TAP1
WNT10B

SSTR2
PKLR
PDE6A
PRLR
SLC11A2
NPR2
MSX2
CD8A
ILK
LAMA1
GJB2
ERCC1
ATP7A
ACTN4
XPA
MTR
TRPM6
TK1
PRNP
PDP1
PAX2
RFC1
PRPS1
PSMB9
HEXA
FDPS
ERCC4
EYA1
F11
PKD1
MSX1
ARRB2
ADH4
FRK
IGFALS
CBX5
GFM1
ATP6V1B1
AGL
CKM
EMD
TAP2
TAT
TFR2
STEAP3
SGSH
NPHS1
RPE65

RAN
SEC24D
TP53BP1
ARSB
CD2AP
CCS
HAMP
IRF6
MBTPS2
LPAR3
LMNB2
ERAP1
CLCNKB
ANK3
ADH1B
TAPBP
TLR6
TMPRSS6
VAV3
TYROBP
TRH
TRHR
TFE3
TLL1
SSTR1
SPG7
PLCE1
SLC25A24
SLC29A3
SF3B4
SI
NARS2
GPNMB
COMMD1
DLX3
AP2S1
LAMP1
ICMT
HPGDS
KIF1A
GNA15
GNRH1
DGKQ
ERCC8
CLDN16
ACTC1
ADH1A
B4GALT7

BANF1
CDH23
TCOF1
NAGLU
USH1C
TWIST2
UBA7
STX16
SUMF1
PLS3
MOCOS
MOCS2
SLC31A1
MTRR
ROBO2
NAGS
ASAH2
GSTZ1
MGAM
CNN1
TKTL1
SSTR5
NPHS2
ATOX1
FCAR
BSND
AFF2
INF2
LEMD3
GCM2
DNAJB11
CRTAP
CAPZB
ONECUT1
SYNE1
PNPLA3
MOCS1
PROP1
NUP153
SCLY
SNX10
SMPD3
B3GALT6
IRX5
LDB1
LRRK1
GHRH
CELSR3

CPNE3
CPZ
CYBRD1
ALX1
ALX4
NBAS
SYNE2
TELO2
PRDM5
SEC24B
PDLIM5
PLEK
PCDH15
NIT2
MTX1
NAT10
RAI1
HEPH
KHDRBS3
LHX8
GOLM1
ASAP2
ZNF23
OSTM1
TBX22
VSX1
SUN1
NYX
RCE1
FEZ1
HGSNAT
DBP
ERAP2
DNAH8
ENAM
ZNF354A
NBR1
SUN2
SPECC1L
SOX8
ST18
FOXE3
COL5A3
KLC4
IFT43
KIAA0319L
IGDCC3
FAM111B

CFHR2
ATP6V0E1
ZSWIM6
THSD4
P3H2
LIN54
LOXHD1
MBD6
H2AX
HJV
FBN3
EPDR1
ZNF346
ONECUT2
VIT
TTI2
SSPN
PPP1R12C
NARS1
FSCB
CEP350
IFITM5
FKBP11
ALX3
PRPSAP2
PDF
ARSH
LARP6
F8A1
ADAM18
ZNF469
TMEM222
MT-CO1
KIAA0895
FAM102A
WHRN
OR2D2
P3H4
BSX
BEX3
TAAR9
CMSS1
RBM33
CCDC154
ZNF98
PLPPR3
DIRC1
FRG2C

VCAN-AS1
CA3-AS1
MFF-DT
CNC2
LOC107652445
LOC106627982
LOC106146152
LOC106050103
LOC106146143
LOC106146144
LOC106146150
LOC106146151
BCL2L1
C4BPA
F2RL2
CCN3
TYK2
ITGA2B
MAP3K1
STAT6
ITGA5
HPRT1
ENTPD1
CD9
DKK2
TNFRSF12A
CD63
ADAM8
THPO
SLC3A2
CD84
KERA
CHRD
OGN
TSPAN4
PRKCD
PCNA
YWHAE
ABCA1
CASP9
MMP19
ABCC4
PRDX2
ANGPTL4
P2RX7
TFDP1
LAMA5
E2F1

PRDX3
SEMA3C
MTDH
USP3
CCL27
SNTB1
GPX1
TXNRD2
CORIN
SLC20A1
SELENOP
PDGFRB
MAP2K1
NTRK2
FLT4
G6PD
GRB2
IRS1
HSPA8
GFAP
HSD3B2
CCNB1
PTGER3
KLF4
SCARB1
COL18A1
FGF5
DLX5
SEMA4A
IL12B
ID1
ITGB7
CMA1
CITED2
ELK1
THBS4
SKIL
HSPA4
NTF3
MT2A
HOXD9
GRP
MARCO
AZU1
RND1
OMD
C1QTNF3
IFI27

A1BG
CALCB
VSIG4
ITGBL1
CPQ
H2BC21
SELENOS
H3C14
MIR194-1
JAK1
PIK3CD
PTPN11
HSPB1
ABCB1
CSNK2A1
CYP3A4
GRIN1
PRKCH
SLC9A1
MYB
RARB
ITGA6
KCNMA1
TF
STK11
RRM2B
EEF2
ALDOA
SLC2A3
SCNN1B
GDNF
MAP3K3
MAP3K11
EPHB1
ESRRA
ADAM9
CAMK2G
CHAT
CDH11
EPHX1
REN
TTN
TYRO3
TFAP2A
C1QA
IRF8
HMGA1
GFPT1

DDX58
EFNB1
CD46
CYBB
ACLY
BAP1
DAG1
VIPR1
SUMO1
MYCN
PTPN2
ITCH
CCNA2
AICDA
TCF7L2
THBS2
SREBF1
MMP15
PTPRB
KCNA2
FZD10
C1QB
CD274
GRK6
HSF1
IDO1
ID2
LAMA4
FABP4
CENPE
C1QC
AGRN
USP14
VIPR2
TPH1
PRF1
NR2C2
PTGDS
SEMA4D
RXFP1
FSCN1
GAB2
B3GAT1
INHA
ITGA9
MAP3K4
HDAC10
GRK5

GSTA4
AGTR2
CLOCK
ACD
CDH13
EEF1A1
DNASE1L3
MDK
TRAP1
TIRAP
SMAD1
PLTP
PLXNB1
PRMT5
PDGFA
PDYN
PHOX2B
RAMP1
TNFRSF14
FOXO4
GRPR
ID3
IFI16
LILRB1
LPIN2
CDKN3
CPA6
DHRS3
CDO1
P2RX3
TREX1
TIE1
TNFRSF10D
TNFRSF18
SETDB1
PCM1
KLF15
IL15RA
CSGALNACT2
CAPZA1
BATF
ATG12
PADI3
G3BP1
BCL3
CCR9
GNG5
EDIL3

BHLHE41
CHRDL1
TRIM2
TERF2
TRAIP
TAB3
TEP1
TPST1
SULF2
SLC9A2
OXT
RCOR1
HPX
IVNS1ABP
DEFB1
ADAM28
BAIAP2L1
TTF2
TNFRSF10C
TNFSF18
MKKS
FBLIM1
C1D
CD53
CD5L
TRIM38
TINAGL1
SRGN
PDIA2
IER3
DEFA5
IL17D
UCN3
TRIB1
TRIB2
PIWIL2
PIWIL4
NLRP6
MYCL
RASGRP4
SAP30BP
ADPRH
HECTD2
DEFA3
DEFA6
CLEC4E
MKX
PRTG

GCNT4
EFS
ZNF277
PTPA
PGPEP1
IL26
LBH
ADM2
CLEC5A
ZNF302
UCN2
TTC38
H3C1
SFMBT2
CNMD
TERC
RBIS
PABIR2
MIR143
MIR124-1
MIR142
MIR19A
MIR216A
MIR19B1
MIR1275
MCM3AP-AS1
MIR608
MIR92A2
MIR519B
MIR382
MIR558
MIR602
MIR203B
CDR1-AS
TALDO1P1
LOC108021846
CSNK1D
PTPN1
CSNK2B
GNB3
PLA2G6
GZMB
MMP11
REST
PAPSS1
COPS5
DDAH2
GABPA

APOM
LY6G5C
FRA1H
CALCRL
MAP3K4-AS1
FSHR
IFNGR1
CASP7
RDX
IDE
MAPKAPK2
TRPC6
TPO
FZD5
MC2R
HSP90AA1
DLG4
RBPJ
TRPC3
PIK3C2A
MSN
ITGA2
KLK3
ITGB6
FGF10
DCC
OPRL1
P2RY2
SLC20A2
ADORA2A
HSPG2
IGF2R
HRH1
EZR
ITGAX
HGS
FGF14
SMURF1
SNCAIP
LBP
CBX3
ENPP2
FOSB
CD70
ITGA11
FGF13
CENPJ
DDB1

MC5R
FOSL2
MIP
TRPC5
PTGDR2
EPO
BRD1
CD276
KCNN1
RHOD
SFRP5
SERPINA7
ITGA10
MRPL17
NOX5
DPT
ACKR1
P4HTM
NUPR1
ABHD16A
DEFB104A
GPANK1
C6orf47
CGAS
CIP2A
DEFB104B
HOTTIP
LINC-PINT
MIR770
FADD
KITLG
IL21R
CNTF
ERRFI1
MDM2
A2M
HNRNPA2B1
IL36B
ELP1
MAP2K2
KIT
PLK1
SQSTM1
NR1H4
NR1H2
KRT18
GNAI1
CASP10

TXNRD1
PNLIP
PRKAB1
PTGIS
IRF3
LIFR
F13A1
CDK1
TBX5
WEE1
CRHR1
EIF2S1
SNAI2
ALPI
SERPINA6
HNRNPD
EHF
DIO1
GAST
ADAMTS16
LAIR2
HEXD
MIR126
HAS2-AS1
GRASLND
EGFR
ATR
JAK3
AXL
CDK2
NTRK3
CDC42
PGR
IRAK1
CYLD
EGLN1
EIF2AK3
MEF2C
VCP
AXIN2
PLD1
ODC1
RHEB
IRAK4
IL2RG
MAPK13
IFIH1
GLI1

TLR7
PIN1
PRMT1
PITX2
PTGIR
PTK2
SDHA
YWHAZ
ATF4
KDM6A
CARM1
GGCX
CLTC
NR4A1
SMAD9
PDE8B
PDK4
NEFH
HELLS
CISH
EIF2AK2
TGFBI
RASGRP2
MAP3K14
IL10RB
GP6
CLEC7A
AMD1
CBFB
EIF2AK4
MDM4
UGT2B7
TNFSF15
SRD5A1
PLAA
PPM1A
PDE4B
S1PR2
CYSLTR1
HSPA1A
GAS6
ABCB7
AGRP
ENPP3
NPPB
UGT1A9
TRAF3IP2
TRADD

RGS4
PCSK7
NTF4
ROM1
RGS10
PTPRK
AREG
ANXA7
H6PD
BNIP3
AIMP1
DUSP5
USP5
TPR
PYY
SEMA7A
RASGRF1
IAPP
LIPF
HNRNPC
BRD3
HCRT
HSPB2
LMOD1
GLRA3
RGS5
RGS7
CD83
ITGAE
MAPRE3
IFNA1
IL1F10
EBI3
CTRL
AP2A1
UXS1
SPAM1
SLC16A4
IRX1
NELL1
EBAG9
UGT1A3
TXNDC5
TSPAN9
CCL17
MADCAM1
CHST4
TET1

PHOSPHO1
PIEZO1
COL20A1
CASC3
TNIP3
TP53AIP1
LILRA5
ENGASE
CNN3
ARL15
RNPC3
PPARGC1B
CD24
HIVEP3
RNASE7
JPH4
GCFC2
CSN1S1
VEGFD
RASL11B
CSRNP1
CCN5
ZBED3
GDPD4
ERV3-1
NAA80
TSBP1
MIR192
MIR499A
MIRLET7A1
MIR191
MIR155HG
SNHG7
MIR181B1
MIR302D
MIR302B
EPB41L4A-AS1
MIR532
MIR1276
SAA3P
UGT1A
TRAP
STQTL2
DDH1
