# Supplementary material for: Exploring the Mechanism of Resveratrol in Reducing the Soft Tissue Damage of Osteoarthritis Based on Network Pharmacology and Experimental Pharmacology
Source: Evid Based Complement Alternat Med. 2021 Oct 4;2021:9931957. doi: 10.1155/2021/9931957 (PMC8505078; doi:10.1155/2021/9931957)
Supplement: Supplementary Materials — Table S1: Resveratrol Potential Targets. Table S2: OA genes. Table S3: Enrichment Analysis of Resveratrol-OA PPI Network. [file 9931957.f1.zip › 9931957.f1/Table S2-revised (2).pdf]

Table S2 Resveratrol-OA Targets

BMP2
STS
ALB
PDPK1
MMP3
PIK3CG
MAPK14
ADAM17
ESR1
GC
MAPK10
MAPK1
CA2
F2
SRC
DPP4
PDE4B
MMP8
FGFR1
PDE4D
AR
SULT2A1
PGF
HSD11B1
PTPN11
AKR1C1
TTR
GSK3B
CDK2
MMP13
ESR2
EGFR
GSTP1
MDM2
PPARA
SHBG
KDR
PTPN1
TGFBR1
TRAPPC3
NR1H3
ELANE
PGR
CYP2C9
HPGDS
NR1H2
MET

DHODH
CCNA2
JAK3
F10
ZAP70
MMP2
HDAC8
SYK
RBP4
SERPINA1
REN
NR3C1
PADI4
HSP90AA1
CTSS
MMP12
ITGAL
S100A9
PLA2G2A
PPARG
DPEP1
NR1H4
XIAP
MAPKAPK2
HMGCR
F11
IGF1
IL2
TEK
MAP2K1
THRA
VDR
KIT
RARB
CTSB
CTSK
PIK3R1
TGFB2
MMP9
PPP1CC
CASP1
PROCR
NFE2L2
APP
PTGS1
PTGS2
CA1
TRPA1

CYP3A4
PIK3CB
RELA
MIF
AHR
ALOX5
ODC1
MAPT
CXCL12
CYP1A1
F3
TYR
MMP1
KDM1A
SIRT1
CYP19A1
ABCB1
PTK2B
ESRRA
ESRRB
IGF1R
ALOX15
LTB4R
HDAC2
VCP
PLAT
BCL2L1
BCL2
CALM1
NOS1
MTOR
IGFBP3
NOS2
NOS3
WEE1
NOX4
LMNA
KCNMA1
ARSA
PRKN
HDAC9
CASP7
CASP3
HTT
NFKB1
NFKB2
PTH1R
NPC1

TP53
HPGD
HIF1A
SMAD2
SMAD3
MEN1
PAK1
RGS4
CYP2D6
NR1I3
NPR2
GAA
MYC
TERT
CD38
CTSD
SIRT2
PRKCA
DUSP1
NFKBIA
FPR2
S1PR1
BAP1
CTSL
ITGA4
C1S
ALPL
CTSG
HSPA8
MMP14
PTPN22
RAC1
NCOA3
STAT3
GNAI1
STAT1
PTGER2
HSPA1A
INS
EIF2AK3
RGS7
TNFSF10
CBFB
JAK2
NPSR1
GLA
MAP3K3
PHOSPHO1

NOD2
NOD1
PKM
KLF5
CTNNB1
OPRK1
PLEC
NLRP1
GBA
MITF
BCL2L11
GAPDH
ALPI
KCNQ1
SENP6
RXFP1
NLRP3
SLC2A3
ABCC2
FOXO3
AKT1
IDE
FURIN
MAP1LC3A
MDM4
ATM
ADRB2
EYA2
TRPV1
TRHR
CCR6
OPRD1
OPRM1
KEAP1
PIN1
PRKAR2B
TLR9
RAN
SMPD1
P4HB
DNMT1
CXCR6
TLR3
SUMO1
TACC3
ERG
NSD2
RIPK2

GNAS
EPAS1
TNFRSF10B
MCOLN1
PDE5A
CYSLTR1
MC5R
MAPK3
PPP3CA
ERBB2
PTPRC
HTR2A
ADORA2A
ADRB1
AGTR2
BDKRB2
CALCR
SLC6A4
TACR1
FLT1
VIPR1
CNR1
CCR2
CCR4
CCR5
CXCR1
CXCR2
EDNRA
HRH1
HRH2
EZH2
UBE2M
TNF
CXCL8
WRN
COPS5
TARDBP
SNCA
S100A4
ERAP1
PRMT1
SELE
UHRF1
PLK1
ADAM10
PRNP
FOXO1
PLA2G3

HSPB1
IL1B
NRP2
SIAE
ATF6
JUN
GALC
CGAS
IFNG
YAP1
SIRPA
CD47
IFNB1
HLA-B
AICDA
