# Supplementary material for: Exploring the Mechanism of Resveratrol in Reducing the Soft Tissue Damage of Osteoarthritis Based on Network Pharmacology and Experimental Pharmacology
Source: Evid Based Complement Alternat Med. 2021 Oct 4;2021:9931957. doi: 10.1155/2021/9931957 (PMC8505078; doi:10.1155/2021/9931957)
Supplement: Supplementary Materials — Table S1: Resveratrol Potential Targets. Table S2: OA genes. Table S3: Enrichment Analysis of Resveratrol-OA PPI Network. [file 9931957.f1.zip › 9931957.f1/Table S3-revised (1).pdf]

## Table S3 Enrichment Analysis of Resveratrol-OA PPI Network

| Category | Term | Description | Count | % | PValue | Genes |
|---|---|---|---|---|---|---|
| **Biological processes** | GO:0001501 | skeletal system c | 32 | 12.4031 | 7.28E-28 | COL11A2, |
| | GO:0043401 | steroid hormone | 21 | 8.139535 | 1.33E-22 | RARG, TH |
| | GO:0030574 | collagen catabol | 20 | 7.751938 | 6.08E-20 | MMP1, CO |
| | GO:0006367 | transcription init | 24 | 9.302326 | 8.37E-17 | RARG, TH |
| | GO:0030198 | extracellular ma | 26 | 10.07752 | 2.32E-16 | COL11A1, |
| | GO:0022617 | extracellular ma | 18 | 6.976744 | 1.09E-15 | MMP1, M |
| | GO:0030199 | collagen fibril or | 13 | 5.03876 | 3.01E-13 | TGFB2, C |
| | GO:0045893 | positive regulati | 35 | 13.56589 | 4.54E-13 | SRC, NR1 |
| | GO:0043066 | negative regulati | 32 | 12.4031 | 2.36E-12 | GSK3B, R |
| | GO:0043627 | response to estro | 14 | 5.426357 | 1.30E-11 | DTYMK, |
| | GO:0001503 | ossification | 15 | 5.813953 | 1.42E-11 | SLC26A2, |
| | GO:0008284 | positive regulati | 31 | 12.0155 | 2.27E-11 | RARG, EC |
| | GO:0007179 | transforming gro | 15 | 5.813953 | 1.01E-10 | SMAD2, T |
| | GO:0006954 | inflammatory res | 27 | 10.46512 | 1.34E-10 | CRP, CXC |
| | GO:0070374 | positive regulati | 19 | 7.364341 | 1.70E-10 | MAP2K1, |
| | GO:0000187 | activation of MA | 15 | 5.813953 | 7.99E-10 | MAP2K1, |
| | GO:0010718 | positive regulati | 10 | 3.875969 | 1.01E-09 | SMAD2, C |
| | GO:0008285 | negative regulati | 26 | 10.07752 | 1.75E-09 | RARG, CX |
| | GO:0030335 | positive regulati | 18 | 6.976744 | 2.92E-09 | TGFB1, SI |
| | GO:0030522 | intracellular rece | 10 | 3.875969 | 4.00E-09 | AR, THRB |
| | GO:0030168 | platelet activatio | 14 | 5.426357 | 1.98E-08 | SYK, PDP |
| | GO:0050728 | negative regulati | 12 | 4.651163 | 2.79E-08 | SMAD3, T |
| | GO:0014068 | positive regulati | 11 | 4.263566 | 4.53E-08 | TGFB2, EI |
| | GO:0002062 | chondrocyte diff | 9 | 3.488372 | 1.01E-07 | BMP2, CO |
| | GO:0007165 | signal transducti | 43 | 16.66667 | 1.10E-07 | ITK, CXC |
| | GO:0048384 | retinoic acid rec | 7 | 2.713178 | 1.80E-07 | RXRB, RA |
| | GO:0050900 | leukocyte migrat | 13 | 5.03876 | 3.30E-07 | MMP1, SF |
| | GO:0042593 | glucose homeost | 12 | 4.651163 | 3.66E-07 | IL6, RBP4 |
| | GO:0018105 | peptidyl-serine p | 13 | 5.03876 | 4.30E-07 | GSK3B, S |
| | GO:0030512 | negative regulati | 10 | 3.875969 | 4.80E-07 | SMAD2, A |
| | GO:0007173 | epidermal growt | 9 | 3.488372 | 1.86E-06 | ADAM17, |
| | GO:0007169 | transmembrane r | 11 | 4.263566 | 1.89E-06 | ITK, ZAP7 |
| | GO:0001958 | endochondral os | 7 | 2.713178 | 2.02E-06 | COL1A1, |
| | GO:0001666 | response to hypo | 14 | 5.426357 | 2.20E-06 | TGFB2, SI |
| | GO:0000165 | MAPK cascade | 17 | 6.589147 | 2.52E-06 | MAP2K1, |
| | GO:0008202 | steroid metaboli | 8 | 3.100775 | 3.38E-06 | SULT2B1, |
| | GO:0007183 | SMAD protein c | 5 | 1.937984 | 3.41E-06 | SMAD2, F |
| | GO:0043410 | positive regulati | 10 | 3.875969 | 3.63E-06 | AR, IL6, E |
| | GO:0060395 | SMAD protein s | 9 | 3.488372 | 4.10E-06 | SMAD2, T |
| | GO:0048015 | phosphatidylinos | 11 | 4.263566 | 4.68E-06 | ERBB4, PI |
| | GO:0051384 | response to gluc | 9 | 3.488372 | 5.88E-06 | IL10, BCH |
| | GO:0010862 | positive regulati | 8 | 3.100775 | 7.24E-06 | TGFB2, SI |
| | GO:0032496 | response to lipo | 13 | 5.03876 | 7.57E-06 | MAOB, TI |
| | GO:0030282 | bone mineralizat | 7 | 2.713178 | 1.06E-05 | BMP2, MN |
| | GO:0032869 | cellular response | 9 | 3.488372 | 2.10E-05 | PDPK1, SI |
| | GO:0014066 | regulation of ph | 9 | 3.488372 | 2.31E-05 | ERBB4, L |

| | | | | | |
|---|---|---|---|---|---|
| GO:0006955 | immune respons | 20 | 7.751938 | 2.34E-05 | IL10, IL1 |
| GO:0045780 | positive regulati | 5 | 1.937984 | 3.28E-05 | SYK, CA2 |
| GO:0010745 | negative regulati | 5 | 1.937984 | 3.28E-05 | CRP, NR1 |
| GO:0060389 | pathway-restrict | 5 | 1.937984 | 3.28E-05 | TGFB2, B |
| GO:0003417 | growth plate car | 4 | 1.550388 | 3.34E-05 | COMP, M |
| GO:0030593 | neutrophil chem | 8 | 3.100775 | 6.07E-05 | TGFB2, C |
| GO:0010887 | negative regulati | 4 | 1.550388 | 6.61E-05 | NR1H2, N |
| GO:0042110 | T cell activation | 7 | 2.713178 | 7.21E-05 | FKBP1A, |
| GO:0071560 | cellular response | 7 | 2.713178 | 9.16E-05 | COL1A1, |
| GO:0043552 | positive regulati | 6 | 2.325581 | 9.38E-05 | TGFB1, E |
| GO:0000122 | negative regulati | 26 | 10.07752 | 9.86E-05 | RARG, TH |
| GO:0032331 | negative regulati | 5 | 1.937984 | 1.04E-04 | RARG, RA |
| GO:0030500 | regulation of bon | 5 | 1.937984 | 1.32E-04 | ANKH, BC |
| GO:0001837 | epithelial to mes | 6 | 2.325581 | 1.48E-04 | GSK3B, T |
| GO:0060021 | palate developm | 8 | 3.100775 | 1.50E-04 | SMAD2, T |
| GO:0048146 | positive regulati | 7 | 2.713178 | 1.59E-04 | CCNA2, T |
| GO:0045416 | positive regulati | 4 | 1.550388 | 1.81E-04 | APOA2, T |
| GO:0070723 | response to chol | 4 | 1.550388 | 1.81E-04 | SMAD2, T |
| GO:0043065 | positive regulati | 15 | 5.813953 | 1.99E-04 | RARG, TC |
| GO:0007184 | SMAD protein i | 4 | 1.550388 | 2.69E-04 | TGFB2, T |
| GO:0050852 | T cell receptor s | 10 | 3.875969 | 4.25E-04 | ITK, ZAP7 |
| GO:0070371 | ERK1 and ERK | 5 | 1.937984 | 4.28E-04 | MAP2K1, |
| GO:0010575 | positive regulati | 5 | 1.937984 | 6.82E-04 | IL1A, TGF |
| GO:0002224 | toll-like recepto | 5 | 1.937984 | 6.82E-04 | CTSK, MA |
| GO:0071347 | cellular response | 7 | 2.713178 | 7.11E-04 | IL6, CXCI |
| GO:0048010 | vascular endothe | 7 | 2.713178 | 7.66E-04 | HSP90AA |
| GO:2000352 | negative regulati | 5 | 1.937984 | 7.87E-04 | IL10, ABL |
| GO:2001275 | positive regulati | 4 | 1.550388 | 8.74E-04 | AKT2, NR |
| GO:0060391 | positive regulati | 4 | 1.550388 | 8.74E-04 | SMAD4, T |
| GO:0006693 | prostaglandin m | 4 | 1.550388 | 8.74E-04 | HPGDS, A |
| GO:0051092 | positive regulati | 9 | 3.488372 | 9.47E-04 | AR, IL6, T |
| GO:0030217 | T cell differentia | 5 | 1.937984 | 0.001028 | ZAP70, LC |
| GO:0071732 | cellular response | 4 | 1.550388 | 0.0011 | CCNA2, C |
| GO:0048701 | embryonic crani | 5 | 1.937984 | 0.001167 | SMAD2, S |
| GO:0071392 | cellular response | 5 | 1.937984 | 0.001167 | IL10, CCN |
| GO:0016477 | cell migration | 10 | 3.875969 | 0.00124 | GSK3B, T |
| GO:0035556 | intracellular sign | 16 | 6.20155 | 0.001274 | SMAD2, C |
| GO:0032909 | regulation of tra | 3 | 1.162791 | 0.00134 | TGFB2, SI |
| GO:0071364 | cellular response | 5 | 1.937984 | 0.001482 | COL1A1, |
| GO:0043491 | protein kinase B | 5 | 1.937984 | 0.001482 | TGFB1, IL |
| GO:0070373 | negative regulati | 6 | 2.325581 | 0.001815 | PTPN1, SM |
| GO:0032722 | positive regulati | 4 | 1.550388 | 0.001988 | IL6, ADAI |
| GO:0042523 | positive regulati | 4 | 1.550388 | 0.001988 | ERBB4, K |
| GO:0060749 | mammary gland | 4 | 1.550388 | 0.001988 | AR, ERBB |
| GO:0050714 | positive regulati | 5 | 1.937984 | 0.002059 | IL1A, TGF |
| GO:0050679 | positive regulati | 6 | 2.325581 | 0.002111 | IL6, TGFB |
| GO:0031622 | positive regulati | 3 | 1.162791 | 0.002212 | IL1B, PTG |
| GO:1990314 | cellular response | 3 | 1.162791 | 0.002212 | CCNA2, T |

| | | | | | | |
|---|---|---|---|---|---|---|
| | GO:0010936 | negative regulati | 3 | 1.162791 | 0.002212 | TGFB2, T( |
| | GO:1905007 | positive regulati | 3 | 1.162791 | 0.002212 | TGFB2, T( |
| | GO:0060336 | negative regulati | 3 | 1.162791 | 0.002212 | NR1H2, N |
| | GO:0006950 | response to stres | 6 | 2.325581 | 0.002272 | HSP90AA |
| | GO:0001523 | retinoid metabol | 6 | 2.325581 | 0.002272 | RBP4, TTI |
| | GO:2001240 | negative regulati | 5 | 1.937984 | 0.002281 | IL1A, COI |
| | GO:0033189 | response to vitar | 4 | 1.550388 | 0.002359 | ARG1, RA |
| | GO:0007597 | blood coagulatic | 4 | 1.550388 | 0.002359 | F9, F10, F |
| | GO:0033280 | response to vitar | 4 | 1.550388 | 0.002359 | TGFB1, B( |
| | GO:0032720 | negative regulati | 5 | 1.937984 | 0.00252 | IL10, GST |
| | GO:0001568 | blood vessel dev | 5 | 1.937984 | 0.00252 | COL1A1, ( |
| | GO:2001237 | negative regulati | 5 | 1.937984 | 0.00252 | AR, SRC, |
| | GO:0038128 | ERBB2 signalin | 5 | 1.937984 | 0.00252 | HSP90AA |
| | GO:0032332 | positive regulati | 4 | 1.550388 | 0.00277 | SMAD3, S |
| | GO:0045672 | positive regulati | 4 | 1.550388 | 0.00277 | CA2, TNF |
| | GO:0043124 | negative regulati | 5 | 1.937984 | 0.003047 | GSTP1, NI |
| | GO:0038096 | Fc-gamma recep | 8 | 3.100775 | 0.003201 | HSP90AA |
| | GO:0017015 | regulation of tra | 4 | 1.550388 | 0.003223 | SMAD2, S |
| | GO:0061037 | negative regulati | 3 | 1.162791 | 0.003284 | TGFB2, FI |
| | GO:0032964 | collagen biosynt | 3 | 1.162791 | 0.003284 | COL1A1, ( |
| | GO:0002740 | negative regulati | 3 | 1.162791 | 0.003284 | IL10, APO |
| | GO:0007182 | common-partner | 3 | 1.162791 | 0.003284 | SMAD2, T |
| | GO:0001957 | intramembranou | 3 | 1.162791 | 0.003284 | COL1A1, |
| | GO:0045599 | negative regulati | 5 | 1.937984 | 0.003645 | IL6, TGFE |
| | GO:0042346 | positive regulati | 4 | 1.550388 | 0.003719 | IL1B, PTG |
| | GO:0046427 | positive regulati | 4 | 1.550388 | 0.004258 | IL10, IL6, |
| | GO:0045453 | bone resorption | 4 | 1.550388 | 0.004258 | SRC, CTS |
| | GO:0071639 | positive regulati | 3 | 1.162791 | 0.004552 | IL1A, IL1I |
| | GO:0032755 | positive regulati | 5 | 1.937984 | 0.004683 | IL1A, IL6, |
| | | | | | | |
| **Cell Components** | GO:0005576 | extracellular reg | 87 | 33.72093 | 4.29E-29 | SERPINA |
| | GO:0005615 | extracellular spa | 74 | 28.68217 | 1.01E-24 | IL1RN, SE |
| | GO:0005578 | proteinaceous ex | 34 | 13.17829 | 9.49E-22 | SERPINA |
| | GO:0005829 | cytosol | 101 | 39.14729 | 2.64E-15 | PNMT, HI |
| | GO:0005788 | endoplasmic reti | 24 | 9.302326 | 3.61E-15 | BCHE, SE |
| | GO:0031012 | extracellular ma | 26 | 10.07752 | 7.22E-13 | AEBP1, H |
| | GO:0070062 | extracellular exo | 84 | 32.55814 | 6.79E-12 | IL1RN, SE |
| | GO:0005581 | collagen trimer | 15 | 5.813953 | 3.88E-11 | MMP1, CO |
| | GO:0031093 | platelet alpha gr | 9 | 3.488372 | 9.36E-07 | CFD, TGF |
| | GO:0031234 | extrinsic compo | 9 | 3.488372 | 4.85E-06 | ITK, ZAP7 |
| | GO:0009986 | cell surface | 23 | 8.914729 | 8.88E-06 | HSP90AA |
| | GO:0043235 | receptor comple | 11 | 4.263566 | 1.24E-05 | RXRA, SM |
| | GO:0000790 | nuclear chromat | 12 | 4.651163 | 9.28E-05 | SMAD2, A |
| | GO:0005737 | cytoplasm | 100 | 38.75969 | 3.23E-04 | IL1RN, NI |
| | GO:0005654 | nucleoplasm | 61 | 23.64341 | 3.61E-04 | RARG, TH |
| | GO:0005925 | focal adhesion | 16 | 6.20155 | 4.43E-04 | MAP2K1, |
| | GO:0005594 | collagen type IX | 3 | 1.162791 | 5.84E-04 | COL9A1, ( |
| | GO:0045121 | membrane raft | 11 | 4.263566 | 6.88E-04 | BACE1, D |

| | | | | | | |
|---|---|---|---|---|---|---|
| | GO:0043234 | protein complex | 16 | 6.20155 | 7.59E-04 | HSP90AA |
| | GO:0090575 | RNA polymeras | 5 | 1.937984 | 0.00113 | SMAD4, F |
| | GO:0036021 | endolysosome lu | 3 | 1.162791 | 0.001158 | CTSK, CT |
| | GO:0005796 | Golgi lumen | 7 | 2.713178 | 0.002341 | ACAN, TC |
| | GO:0005911 | cell-cell junction | 9 | 3.488372 | 0.003033 | ITK, ZAP7 |
| | GO:0005886 | plasma membrar | 77 | 29.84496 | 0.004931 | GSK3B, II |
| | | | | | | |
| **Molecular Function** | GO:0003707 | steroid hormone | 24 | 9.302326 | 1.06E-27 | RARG, TH |
| | GO:0005201 | extracellular ma | 17 | 6.589147 | 2.75E-15 | COL11A1, |
| | GO:0004879 | RNA polymeras | 14 | 5.426357 | 2.85E-15 | NR1H2, N |
| | GO:0008270 | zinc ion binding | 51 | 19.76744 | 1.76E-11 | RARG, TH |
| | GO:0008144 | drug binding | 14 | 5.426357 | 1.08E-10 | HSP90AB |
| | GO:0004713 | protein tyrosine | 17 | 6.589147 | 1.80E-10 | MAP2K1, |
| | GO:0005125 | cytokine activity | 18 | 6.976744 | 1.54E-09 | IL10, TGF |
| | GO:0004222 | metalloendopept | 15 | 5.813953 | 1.72E-09 | MME, MM |
| | GO:0005496 | steroid binding | 9 | 3.488372 | 4.34E-09 | AR, SULT |
| | GO:0005518 | collagen binding | 11 | 4.263566 | 2.09E-08 | COMP, SM |
| | GO:0005524 | ATP binding | 52 | 20.15504 | 2.75E-08 | GSK3B, IT |
| | GO:0005515 | protein binding | 176 | 68.21705 | 6.78E-08 | IL1RN, RC |
| | GO:0034713 | type I transformi | 6 | 2.325581 | 9.25E-08 | SMAD2, F |
| | GO:0043565 | sequence-specifi | 27 | 10.46512 | 9.51E-08 | RARG, TH |
| | GO:0004672 | protein kinase ac | 22 | 8.527132 | 1.42E-07 | GSK3B, M |
| | GO:0004716 | receptor signalir | 6 | 2.325581 | 1.83E-07 | SYK, ERB |
| | GO:0046332 | SMAD binding | 9 | 3.488372 | 2.30E-07 | SMAD2, F |
| | GO:0003708 | retinoic acid rec | 5 | 1.937984 | 2.55E-07 | RARG, RX |
| | GO:0008237 | metallopeptidase | 11 | 4.263566 | 3.94E-07 | ADAMTS |
| | GO:0030020 | extracellular ma | 5 | 1.937984 | 7.57E-07 | COL2A1, |
| | | | | | | |
| **Signaling Pathway** | hsa04151 | PI3K-Akt signal | 39 | 15.11628 | 2.72E-13 | GSK3B, H |
| | hsa04068 | FoxO signaling | 25 | 9.689922 | 3E-13 | PIK3R1, E |
| | hsa04380 | Osteoclast differ | 22 | 8.527132 | 9.07E-11 | TGFB2, M |
| | hsa05323 | Rheumatoid arth | 17 | 6.589147 | 2.63E-09 | TGFB2, T |
| | hsa04350 | TGF-beta signal | 15 | 5.813953 | 8.57E-08 | SMAD2, T |
| | hsa03320 | PPAR signaling | 13 | 5.03876 | 3.28E-07 | PDPK1, M |
| | hsa04660 | T cell receptor s | 15 | 5.813953 | 7.97E-07 | IL10, GSK |
| | hsa04620 | Toll-like recepto | 14 | 5.426357 | 8.99E-06 | MAP2K1, |
| | hsa04010 | MAPK signaling | 22 | 8.527132 | 9.86E-06 | TGFB2, M |
| | hsa04668 | TNF signaling p | 14 | 5.426357 | 9.98E-06 | MAP2K1, |
| | hsa04064 | NF-kappa B sign | 12 | 4.651163 | 3.36E-05 | ZAP70, C |
| | hsa04370 | VEGF signaling | 10 | 3.875969 | 5.15E-05 | MAP2K1, |
| | hsa04066 | HIF-1 signaling | 12 | 4.651163 | 8.44E-05 | IL6, MAP |
| | hsa04060 | Cytokine-cytoki | 18 | 6.976744 | 0.000561 | IL10, TGF |
| | hsa04920 | Adipocytokine s | 9 | 3.488372 | 0.000808 | RXRB, MA |
| | hsa04150 | mTOR signaling | 8 | 3.100775 | 0.001238 | PDPK1, A |
| | hsa04152 | AMPK signaling | 11 | 4.263566 | 0.002665 | CCNA2, P |
| | hsa04062 | Chemokine sign | 13 | 5.03876 | 0.006948 | GSK3B, IT |
| | hsa04662 | B cell receptor s | 7 | 2.713178 | 0.013722 | GSK3B, M |
| | hsa04640 | Hematopoietic c | 7 | 2.713178 | 0.038027 | IL1A, IL6, |

| Fold Enr | FDR |
|---|---|
| 15.38125 | 1.66E-24 |
| 24.26089 | 1.51E-19 |
| 20.57843 | 4.62E-17 |
| 10.39752 | 4.77E-14 |
| 8.735334 | 1.06E-13 |
| 15.59628 | 4.15E-13 |
| 21.95033 | 9.81E-11 |
| 4.475309 | 1.29E-10 |
| 4.631278 | 5.98E-10 |
| 14.18329 | 2.95E-09 |
| 12.34706 | 2.95E-09 |
| 4.380645 | 4.30E-09 |
| 10.73657 | 1.65E-08 |
| 4.691231 | 2.03E-08 |
| 7.149535 | 2.28E-08 |
| 9.231446 | 7.92E-08 |
| 19.95484 | 9.40E-08 |
| 4.323549 | 1.53E-07 |
| 6.441944 | 2.46E-07 |
| 17.32921 | 3.26E-07 |
| 8.016641 | 1.41E-06 |
| 10.00268 | 1.87E-06 |
| 11.14401 | 2.79E-06 |
| 15.19638 | 6.04E-06 |
| 2.438925 | 6.43E-06 |
| 25.60871 | 1.00E-05 |
| 7.016908 | 1.79E-05 |
| 7.823879 | 1.94E-05 |
| 6.848502 | 2.23E-05 |
| 10.28922 | 2.43E-05 |
| 10.58319 | 8.63E-05 |
| 7.545425 | 8.63E-05 |
| 17.72911 | 9.02E-05 |
| 5.359964 | 9.63E-05 |
| 4.272774 | 1.09E-04 |
| 12.25135 | 1.32E-04 |
| 41.15686 | 1.32E-04 |
| 8.129751 | 1.38E-04 |
| 9.559013 | 1.53E-04 |
| 6.833592 | 1.72E-04 |
| 9.117828 | 2.13E-04 |
| 10.97516 | 2.58E-04 |
| 5.219895 | 2.65E-04 |
| 13.55755 | 3.68E-04 |
| 7.696868 | 6.55E-04 |
| 7.59819 | 7.11E-04 |

| | |
|---|---|
| 3.128313 | 7.12E-04 |
| 25.3273 | 9.36E-04 |
| 25.3273 | 9.36E-04 |
| 25.3273 | 9.36E-04 |
| 52.68078 | 9.41E-04 |
| 7.981937 | 0.001668 |
| 43.90065 | 0.001795 |
| 9.807593 | 0.001912 |
| 9.407283 | 0.002319 |
| 12.74535 | 0.002349 |
| 2.377952 | 0.002442 |
| 19.36794 | 0.002555 |
| 18.29194 | 0.00308 |
| 11.62076 | 0.003408 |
| 6.931682 | 0.003419 |
| 8.536238 | 0.003584 |
| 32.92549 | 0.003895 |
| 32.92549 | 0.003895 |
| 3.292549 | 0.00425 |
| 29.2671 | 0.005372 |
| 4.449391 | 0.008066 |
| 13.71895 | 0.008066 |
| 12.19463 | 0.011272 |
| 12.19463 | 0.011272 |
| 6.49235 | 0.011496 |
| 6.402179 | 0.012298 |
| 11.7591 | 0.012541 |
| 20.26184 | 0.013654 |
| 20.26184 | 0.013654 |
| 20.26184 | 0.013654 |
| 4.456081 | 0.01468 |
| 10.97516 | 0.015733 |
| 18.81457 | 0.016615 |
| 10.62113 | 0.017385 |
| 10.62113 | 0.017385 |
| 3.828545 | 0.018358 |
| 2.614431 | 0.018747 |
| 49.38824 | 0.019099 |
| 9.977421 | 0.020599 |
| 9.977421 | 0.020599 |
| 6.81217 | 0.024489 |
| 15.49435 | 0.0259 |
| 15.49435 | 0.0259 |
| 15.49435 | 0.0259 |
| 9.145969 | 0.026669 |
| 6.585098 | 0.027197 |
| 39.51059 | 0.027705 |
| 39.51059 | 0.027705 |

| | |
|---|---|
| 39.51059 | 0.027705 |
| 39.51059 | 0.027705 |
| 39.51059 | 0.027705 |
| 6.477146 | 0.028113 |
| 6.477146 | 0.028113 |
| 8.898781 | 0.028113 |
| 14.63355 | 0.028458 |
| 14.63355 | 0.028458 |
| 14.63355 | 0.028458 |
| 8.664603 | 0.029611 |
| 8.664603 | 0.029611 |
| 8.664603 | 0.029611 |
| 8.664603 | 0.029611 |
| 13.86336 | 0.031473 |
| 13.86336 | 0.031473 |
| 8.231373 | 0.03422 |
| 4.148093 | 0.034667 |
| 13.1702 | 0.034667 |
| 32.92549 | 0.034667 |
| 32.92549 | 0.034667 |
| 32.92549 | 0.034667 |
| 32.92549 | 0.034667 |
| 32.92549 | 0.034667 |
| 7.839402 | 0.037602 |
| 12.54304 | 0.037849 |
| 11.97291 | 0.041312 |
| 11.97291 | 0.041312 |
| 28.22185 | 0.043426 |
| 7.316776 | 0.044493 |

| | |
|---|---|
| 3.83181 | 1.15E-26 |
| 3.895603 | 1.35E-22 |
| 8.996109 | 8.48E-20 |
| 2.160471 | 1.77E-13 |
| 8.863813 | 1.94E-13 |
| 6.228626 | 3.23E-11 |
| 2.118991 | 2.60E-10 |
| 11.5615 | 1.30E-09 |
| 11.60354 | 2.79E-05 |
| 9.385214 | 1.30E-04 |
| 3.009117 | 2.16E-04 |
| 6.141855 | 2.78E-04 |
| 4.408943 | 0.001913 |
| 1.357919 | 0.006192 |
| 1.553714 | 0.006448 |
| 2.901709 | 0.007425 |
| 70.91051 | 0.00921 |
| 3.786483 | 0.010243 |

| | |
|---|---|
| 2.753806 | 0.01071 |
| 10.74402 | 0.014773 |
| 53.18288 | 0.014773 |
| 5.170558 | 0.028522 |
| 3.710433 | 0.035343 |
| 1.324948 | 0.053651 |
| | |
| 28.15064 | 6.01E-25 |
| 16.6663 | 5.39E-13 |
| 25.5441 | 5.39E-13 |
| 2.865634 | 2.51E-09 |
| 12.09984 | 1.23E-08 |
| 8.395805 | 1.71E-08 |
| 6.717766 | 1.22E-07 |
| 8.719225 | 1.22E-07 |
| 21.89494 | 2.74E-07 |
| 12.04222 | 1.19E-06 |
| 2.28469 | 1.42E-06 |
| 1.31594 | 3.21E-06 |
| 43.78988 | 3.86E-06 |
| 3.423726 | 3.86E-06 |
| 4.025254 | 5.38E-06 |
| 39.41089 | 6.49E-06 |
| 13.74799 | 7.68E-06 |
| 65.68482 | 8.06E-06 |
| 8.920161 | 1.18E-05 |
| 54.73735 | 2.15E-05 |
| | |
| 3.927404 | 2.28E-11 |
| 6.481796 | 2.28E-11 |
| 5.834606 | 2.58E-09 |
| 6.711605 | 4.62E-08 |
| 6.204004 | 1.03E-06 |
| 6.741067 | 3.12E-06 |
| 5.211364 | 6.99E-06 |
| 4.588622 | 6.03E-05 |
| 3.02108 | 6.32E-05 |
| 4.545738 | 6.32E-05 |
| 4.792059 | 0.000166 |
| 5.695479 | 0.000235 |
| 4.342803 | 0.00037 |
| 2.573513 | 0.002122 |
| 4.466883 | 0.00271 |
| 4.792059 | 0.003878 |
| 3.107046 | 0.007411 |
| 2.428234 | 0.018003 |
| 3.524594 | 0.03359 |
| 2.795367 | 0.082573 |
